# The mutation of Transportin 3 gene that causes limb girdle muscular dystrophy 1F induces protection against HIV-1 infection

**DOI:** 10.1371/journal.ppat.1007958

**Published:** 2019-08-29

**Authors:** Sara Rodríguez-Mora, Flore De Wit, Javier García-Perez, Mercedes Bermejo, María Rosa López-Huertas, Elena Mateos, Pilar Martí, Susana Rocha, Lorena Vigón, Frauke Christ, Zeger Debyser, Juan Jesús Vílchez, Mayte Coiras, José Alcamí

**Affiliations:** 1 AIDS Immunopathogenesis Unit, National Center of Microbiology, Instituto de Salud Carlos III, Madrid, Spain; 2 Molecular Virology and Gene Therapy, Department of Pharmaceutical and Pharmacological Sciences, KU Leuven, Flanders, Belgium; 3 Department of Infectious Diseases, Hospital Ramón y Cajal, Alcalá de Henares University, Instituto Ramón y Cajal de Investigación Sanitaria (IRYCIS), Madrid, Spain; 4 Neuromuscular Diseases Unit, Neurology Department, Hospital Universitari i Politècnic La Fe, Valencia, Spain; 5 Laboratory for Photochemistry and Spectroscopy, Molecular Imaging and Photonics, Department of Chemistry, KU Leuven, Flanders, Belgium; 6 Centro de Investigación Biomédica en Red de Enfermedades Raras (CIBERER), Spain; 7 Infectious Diseases Unit, IBIDAPS, Hospital Clínic, University of Barcelona, Spain; Fred Hutchinson Cancer Research Center, UNITED STATES

## Abstract

The causative mutation responsible for limb girdle muscular dystrophy 1F (LGMD1F) is one heterozygous single nucleotide deletion in the stop codon of the nuclear import factor Transportin 3 gene *(TNPO3*). This mutation causes a carboxy-terminal extension of 15 amino acids, producing a protein of unknown function (TNPO3_mut) that is co-expressed with wild-type TNPO3 (TNPO3_wt). TNPO3 has been involved in the nuclear transport of serine/arginine-rich proteins such as splicing factors and also in HIV-1 infection through interaction with the viral integrase and capsid. We analyzed the effect of TNPO3_mut on HIV-1 infection using PBMCs from patients with LGMD1F infected ex vivo. HIV-1 infection was drastically impaired in these cells and viral integration was reduced 16-fold. No significant effects on viral reverse transcription and episomal 2-LTR circles were observed suggesting that the integration of HIV-1 genome was restricted. This is the second genetic defect described after CCR5Δ32 that shows strong resistance against HIV-1 infection.

## Author summary

TNPO3 has been described as a key factor in the infection by the human immunodeficiency virus (HIV-1), the causative agent of AIDS. In 2013, a relationship between a genetic defect in *TNPO3* gene and a rare muscle disease named Limb Girdle Muscular Dystrophy 1F (LGMD1F), with an autosomal dominant transmission, was discovered. LGMD1F patients show a heterozygous single nucleotide deletion in the *TNPO3* gene that generates a TNPO3_mut protein. Our results demonstrate that cells from patients with this mutation in TNPO3 are resistant to HIV-1 infection in vitro. We are faced with an in vivo situation in which the genetic defect that causes this rare disease confers resistance to HIV infection. Therefore, *TNPO3* mutation represents a natural model to understand the pathogenesis of both diseases. Cells from LGMD1F patients can be used to understand the mechanisms of action of TNPO3 in HIV infection and to design new therapeutic strategies for the treatment of both diseases. The use of HIV-1 as a methodological tool will permit a better understanding of the physiopathological mechanisms derived from the mutation in TNPO3 that causes the muscle disease.

## Introduction

Productive HIV-1 infection requires the interaction with cellular co-factors at virtually all the steps of the viral replication cycle [1]. Viral entry depends on fusion of viral and cellular membranes through successive interactions with CD4 receptor combined with CXC chemokine receptor type 4 (CXCR4) or CC chemokine receptor type 5 (CCR5) [2]. Once the core is released into the cytosol, the reverse transcriptase converts the viral RNA genome into a double-stranded copy DNA (cDNA) and the capsid (CA) uncoating process is initiated. HIV-1 cDNA gains access to the nucleus through the cellular nuclear transport machinery located at the nuclear pore, in the form of a pre-integration complex (PIC). These PICs consist of viral cDNA and other HIV-1 components like integrase (IN), matrix, nucleocapsid, CA and viral protein R (Vpr), as well as various host proteins, such as the high mobility group protein B1 (HMGB1), barrier to autointegration factor 1 (BAF1), lamina-associated polypeptide 2α (LAP2α) and lens-epithelium derived growth factor (LEDGF/p75) [3–7]. Several cellular import factors, including importin-7, importin-α3 and Transportin 3 (TNPO3, also called TRN-SR2) have also been involved in HIV-1 nuclear import [8]. Apart from its implication in nuclear import of the viral PIC, it has been confirmed that N-terminal end of TNPO3 protein act as a direct binding partner of HIV-1 IN [9]. Interaction with the viral CA has also been documented [10,11] and nearly 30 CA-mutants able to modify HIV-1 dependence on TNPO3 have been described [12].

TNPO3 is a member of the karyopherin β superfamily of proteins [13,14] that imports into the nucleus mostly serine/arginine (SR)-rich proteins. Within these proteins are essential pre-mRNA splicing factors such as serine/arginine-rich splicing factor 1 (SRSF1), SR-rich splicing factor 2 (SRSF2, also known as SC35) and cleavage and polyadenylation-specific factor 6 (CPSF6) [15,16]. The interaction between HIV-1 CA and CPSF6 impedes interferon (IFN)-mediated innate responses, allowing HIV-1 to escape from immune sensing and favouring infection. In fact, HIV-1 virions carrying CA mutation N74D that cannot interact with CPSF6 trigger innate sensors that induce an antiviral state against HIV-1 infection in macrophages [17]. Moreover, as HIV-1 is highly dependent on the cellular splicing machinery [18], modifications in TNPO3-mediated nuclear import may indirectly affect HIV-1 replication through changes in the post-transcriptional mRNA maturation [19–22]. TNPO3 has been identified as a HIV-1 co-factor in two independent genome-wide siRNA screens [23,24] and as a specific binding partner of HIV-1 IN in a yeast two-hybrid screen [25]. These results support the idea that TNPO3 may be essential for HIV-1 life cycle along with other fundamental proteins such as CPSF6. However, its precise role during HIV-1 nuclear import and viral integration is not fully understood [26].

LGMDs comprise a group of genetically heterogeneous disorders characterized by a progressive and predominantly proximal muscle weakness with histological signs of muscle degeneration and regeneration [27]. In 2001, a novel form of LGMD classified as LGMD1F was reported, affecting 32 individuals in one large Spanish kindred spanning six generations [28]. The genetic defect of this autosomal recessive disease was identified as a single adenosine nucleotide deletion in TAG stop codon of one allele of *TNPO3* gene, common to both protein isoforms encoded by this gene. As a result, the cells from these patients may synthesize both TNPO3_wt and TNPO3_mut proteins forms, being TNO3P-mut an extended form of TNPO3 with fifteen additional amino acids in the C-terminal end. Because the cargo-binding domain of TNPO3 resides in this part of the molecule [29], this function might be altered in the mutated protein [30,31]. Being TNPO3 a co-factor of HIV-1 replication [23–25], the susceptibility to HIV-1 infection of peripheral blood mononuclear cells (PBMCs) isolated from LGMD1F patients was analyzed. Our data revealed that the mutation of *TNPO3* present in patients with LGMD1F protected PBMCs from HIV-1 infection. Therefore, this is the second genetic defect described so far after CCR5-Δ32 deletion [32,33] that is able to confer resistance to HIV-1 infection.

## Results

### Clinical characteristics of LGMD1F patients

**Fig 1:**
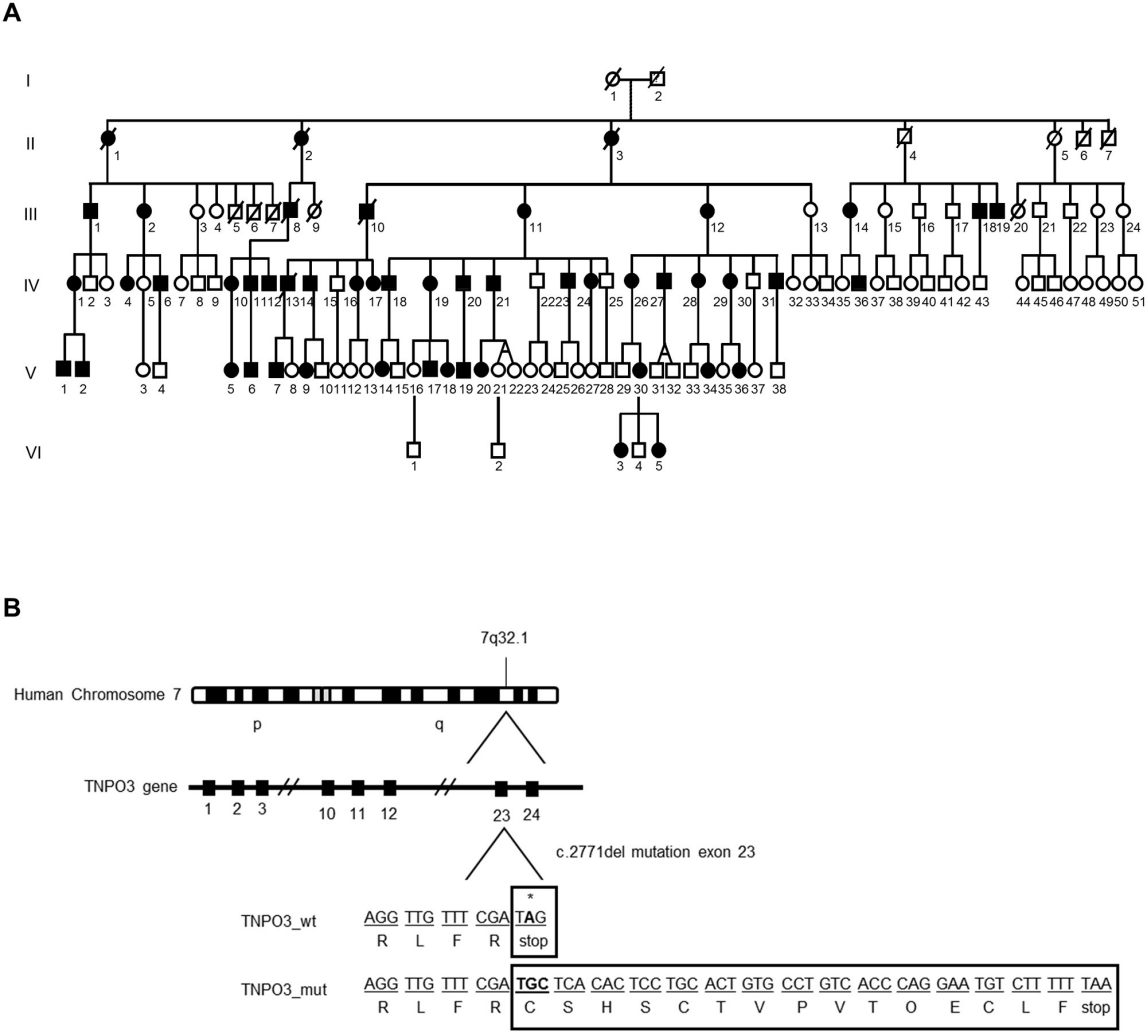
Pedigree and mutation in LGMD1F patients. **(A)** Segregation of *TNPO3* microdeletion (c.2771del). Members of one large Spanish kindred spanning six generations derived from one affected common ancestor. Male and female are indicated by squares and circles, respectively; LGMD1F affected individuals are represented by full filled symbols; and dead individuals are shown by crossed out symbols. **(B)** Schematic representation of the microdeletion in *TNPO3* gene. LGMD1F patients display a mutation located on the long arm (q) of human chromosome 7 (7q32.1). This mutation is a heterozygous single nucleotide deletion (c.2771del) located at exon 23 of *TNPO3* gene, which encodes a 15 amino acid extension of the C-terminus of this protein.

Twenty-three patients with LGMD1F were recruited for this study. All these patients belong to a Spanish/Italian family that shares a common old ancestor born in south-eastern Spain [28], specifically to generations III, IV and V in the family tree (Fig 1A). These patients have been closely followed up at the University Hospital La Fe (Valencia, Spain) and show a wide variety of clinical features (Table 1). Most patients included in this study presented onset symptoms such as difficulties in climbing stairs, rising from sitting, running or fatigue. Thirteen patients showed scapula-humeral and pelvic-femoral weakness and eight of them also presented hand and leg weakness and/or atrophy. The rest of patients showed pelvic-femoral weakness, hand atrophy and leg weakness. Two patients remained asymptomatic when this study was performed. Only three patients presented with grades > 6 in the Vignos score, which is given to individuals who need a long leg brace for walking or standing. Eight patients were graded as 3–4 in the Brooke score and were unable to elevate their shoulders [34,35]. Average levels of creatine kinase (CK) were 3.2-fold higher than the normal range. This human disease is caused by a deletion in the long arm of chromosome 7 (7q32.1) which compromises the *TNPO3* gene. LGMD1F patients show a heterozygous single nucleotide deletion (c.2771del) in exon 23 that generates a 15 amino acid extension of the C-terminus of the protein (Fig 1B).

**Table 1:** Clinical features of LGMD1F patients. Twenty-three LGMD1F patients from three generations were included in this study.

Patient ID	Age at onset	Proximal weakness /atrophy	Distal weakness /atrophy	Brook scale	Vignos scale	Age stage 4 Vignos scale	Serum CK
**III.1**	NA	ASYMPTOMATIC	ASYMPTOMATIC	1	0	NA	NA
**III.12**	40	SH,PF	HAW / LW	3	6	48	350
**IV.1**	NA	ASYMPTOMATIC	ASYMPTOMATIC	NA	0	NA	NA
**IV.16**	16	SH, PF	HAW / LW	2	4	30	175
**IV.17**	10	SH, PF	HAW / LW	2	2	NA	140
**IV.19**	8	SH, PF	HAW / LW	2	3	NA	190
**IV.20**	7	SH, PF	HAW / LW	3	4	30	204
**IV.24**	11	SH, PF	HAW / LW	3	5	12	220
**IV.26**	5	SH, PF	HA / LW	4	7	20	295
**IV.27**	40	SH, PF	HA / LW	2	3	NA	210
**IV.28**	20	SH, PF	HA / LW	2	3	NA	NA
**IV.29**	7	SH, PF	HA / LW	2	3	NA	NA
**IV.31**	18	SH, PF	HAW / LW	2	4	31	744
**V.1**	12	PF	HA / LW	4	4	25	NA
**V.2**	3	PF	HA / LW	4	4	18	NA
**V.5**	NA	NA	NA	NA	NA	NA	NA
**V.9**	14	PF	HA / LW	1	1	NA	NA
**V.13**	NA	NA	NA	NA	NA	NA	NA
**V.18**	14	PF	NA	1	4	20	NA
**V.19**	1	SH, PF	HAW / LW	4	9	15	800
**V.30**	1	SH, PF	HA / LW	3	4	25	NA
**V.34**	7	PF	HA / LW	1	2	NA	220
**V.36**	1	PF	HA / LW	1	3	NA	140

SH: Scapular-humeral; PF: Pelvic-femoral; HAW: Hand atrophy and weakness; LW: Leg weakness; HA: Hand atrophy; CK: creatine kinase; NA: Not available.

### Allelic discrimination of wt and mut *TNPO3* gene

**Fig 2:**
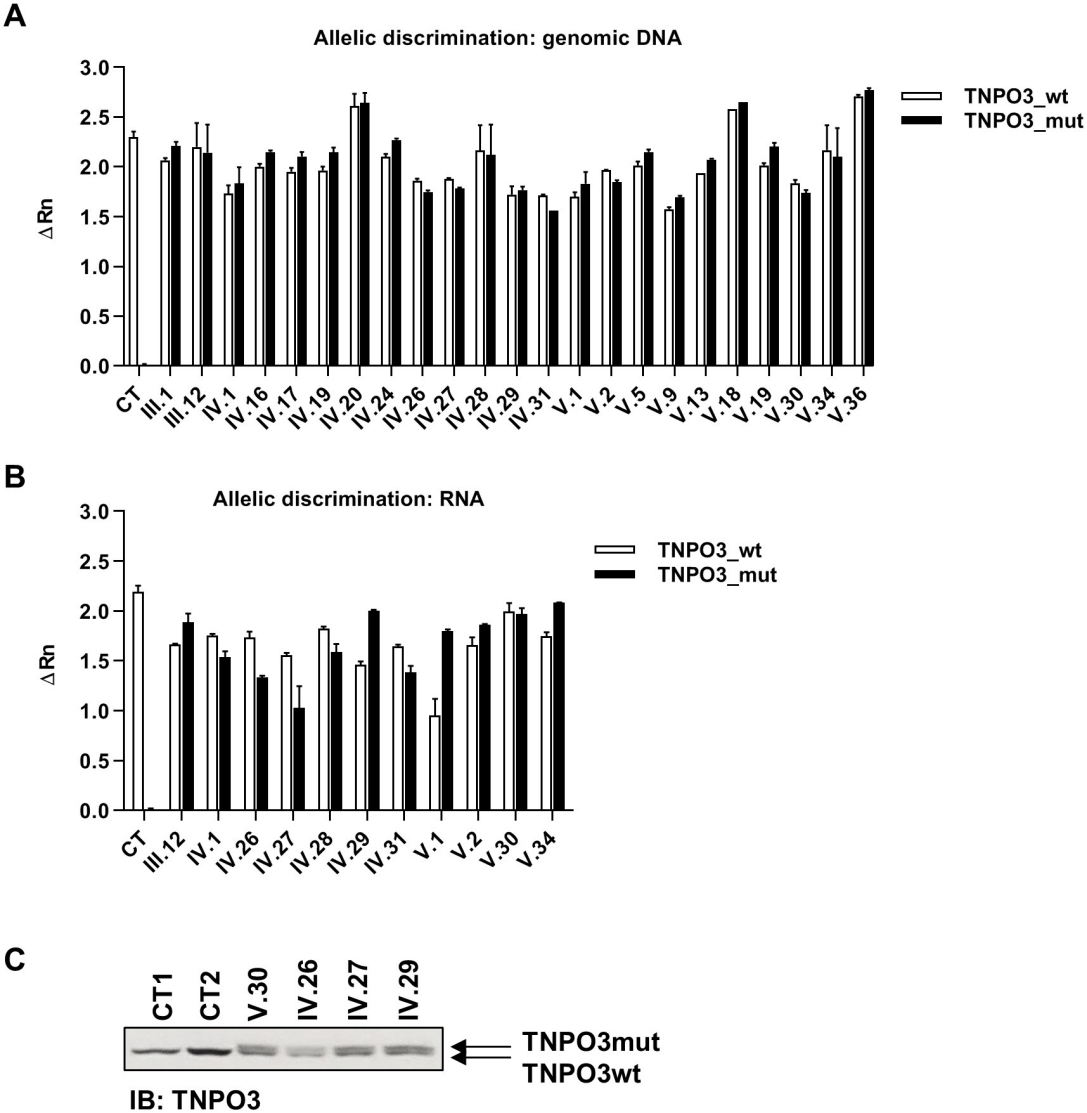
Analysis of the expression of *TNPO3*_wt and *TNPO3*_mut forms. **(A)** Analysis by SNPs genotyping of *TNPO3*_wt and *TNPO3*_mut alleles rate in genomic DNA and **(B)** gene expression in total mRNA obtained from resting PBMCs of LGMD1F patients and healthy controls. **(C)** Analysis by immunoblotting of protein levels of both TNPO3_wt and TNPO3_mut forms in LGMD1F patients and healthy controls. Individual results of duplicates for each LGMD1F patient and mean of all results from healthy controls are represented as a bar graph ± the standard error of the mean (SEM).

The expression pattern of wt and mut variants of TNPO3 was analysed by RT-qPCR in PBMCs isolated from all LGMD1F patients and compared to twenty-seven healthy donors (labelled as CT). All patients revealed co-dominant expression of each allele (Fig 2A and 2B), in comparison with dominant expression of *TNPO3_wt* gene in healthy individuals. In order to know whether the longer protein encoded by *TNPO3_mut* allele was co-expressed with *TNPO3_wt* allele, protein extracts from four patients and two healthy controls were analyzed by immunoblotting using an antibody against TNPO3 that recognized both forms of the protein. Similar levels of TNPO3_wt and TNPO3_mut isoforms were observed in LGMD1F patients, whereas only one band corresponding to TNPO3_wt was observed in healthy controls (Fig 2C).

### Expression of surface and activation markers

The expression levels of the HIV-1 receptor CD4 and the co-receptors CCR5 and CXCR4 were analyzed by flow cytometry in order to exclude an expression defect in cells from LGMD1F patients. No significant difference with healthy controls was found (S1A Fig). PBMCs from LGMD1F patients and controls were then activated with antiCD3, CD28 and IL2 for 48 hours. The expression of activation markers CD25 and HLA-DR was also analyzed but no significant difference was observed (S1B Fig), thus excluding an activation defect influencing cellular susceptibility to HIV-1 infection.

### Localization of CPSF6 protein in LGMD1F patients

TNPO3 is involved in nuclear import of splicing factors such as ASF/SF2, SC35 and CPSF6 [29]. Because it has been shown that TNPO3 knockdown induces the accumulation of CPSF6 –a predominantly nuclear protein- in the cytoplasm [12] we analyzed if the mutant form of TNPO3 from LGMD1F patients resulted in relocalization of CPSF6, a predominantly nuclear protein, to the cytosol. We observed relatively equal levels of CPSF6 protein expression in the nucleus, but PBMCs from LGMD1F patients showed higher level of cytoplasmatic CPSF6 (S2 Fig).

### Resistance to HIV-1 infection in CD4+ T cells from patients with LGMD1F

**Fig 3:**
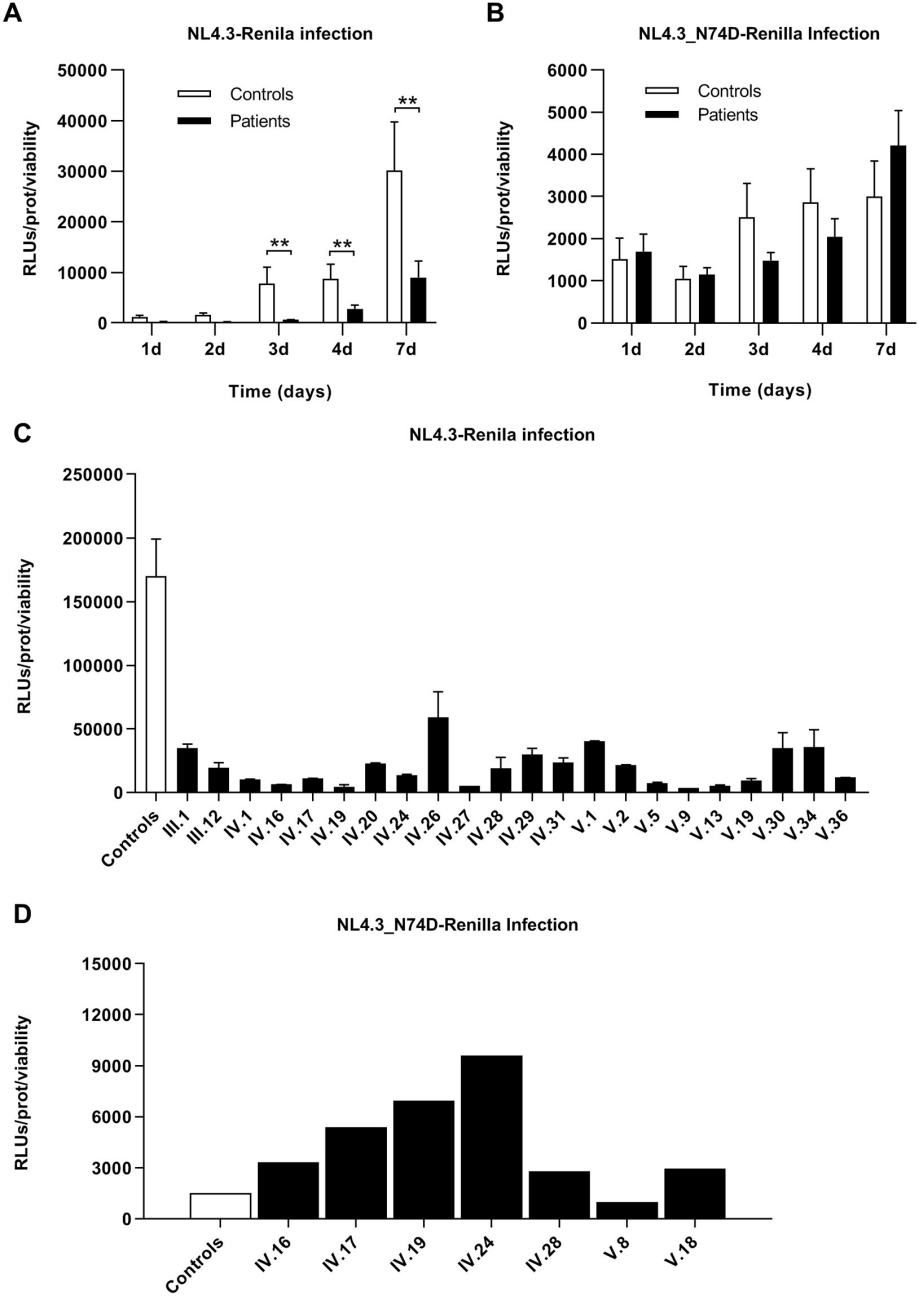
Susceptibility to HIV-1 infection of PBMCs from LGMD1F patients. Activated PBMCs of controls and LGMD1F patients were infected with NL4.3-Renilla and NL4.3_N74D-Renilla to follow a kinetics of viral infection at 1,2,3,4 and 7 days. Analysis of RLUs post infection were measured and the values were normalized for total protein and viability (**A** and **B**, respectively). The analysis of the infection with NL4.3-Renilla shows significant reduction in infection at prolonged replication time (3–7 days **p < 0.01) whereas no significant difference was observed for NL4.3_N74D-Renilla infection at any day post infection. Analysis by chemiluminescence of the production of Renilla (RLUs) in PBMCs from LGMD1F patients and healthy controls infected ex vivo with NL4.3-Renilla (**C**) and NL4.3_N74D-Renilla (**D**) for 5 days. In panel C triplicates for each LGMD1F patient and mean of all results from healthy controls are represented as a bar graph ± the standard error of the mean (SEM). The RLUs mean value in NL4.3-Renilla infection for controls was 159902.946 ± 27875.422 and 26068.964 ± 3545.740 for LGMD1F patients (****p < 0.0001). In panel D, single infection experiments of individual patients are shown. The RLUs mean value in NL4.3_N74D-Renilla infection for controls was 1500.656 ± 160.410 and 4565.722 ± 757.710 for LGMD1F patients (***p < 0.001).

In order to evaluate the susceptibility to HIV-1 infection, we analyzed the kinetics of viral replication in activated PBMCs isolated from seven LGMD1F patients and seven healthy individuals. PBMCs were infected ex vivo by spinoculation with NL4.3-Renilla and NL4.3_N74D-Renilla, a CA mutant in which the nuclear import is independent of TNPO3 [17]. The production of Renilla was measured in cell lysates several days post-infection as relative light units (RLUs). The values were normalized with the total protein taking into account the cell viability during infection. There were no significant differences in cell survival after infection with either virus (S3A and S3B Fig, respectively). Low HIV-1 replication was observed in PBMCs from LGMD1F patients 3–7 days after infection with NL4.3-Renilla (Fig 3A) but no significant difference in the replication of NL4.3_N74D-Renilla virus was observed (Fig 3B).

To confirm these results, activated PBMCs isolated from twenty-two LGMD1F patients and twenty-seven healthy individuals were infected ex vivo with NL4.3-Renilla strain by spinoculation. The production of Renilla was measured in cell lysates 5 days post-infection and results were normalized with total protein and viability. Average virus replication in all LGMD1F patients was reduced 18-fold compared with controls (**** *p*<0.0001) (Fig 3C).

In order to determine whether the impairment in HIV-1 infection was due to the presence of TNPO3_mut in LGMD1F patients and not to other potential restrictive activity, PBMCs isolated from seven LGMD1F patients and seven healthy donors were activated and infected in vitro with NL4.3_N74D-Renilla (Fig 3D). In contrast to the results obtained using a wt HIV-1 clone, reduction in infectivity was observed between LGMD1F and control patients. On the contrary, an increase in HIV-1 replication was observed in six out of seven patients in comparison with controls (p<0.001).

### Dissection of HIV-1 replication cycle in PBMCs from LGMD1F patients

**Fig 4:**
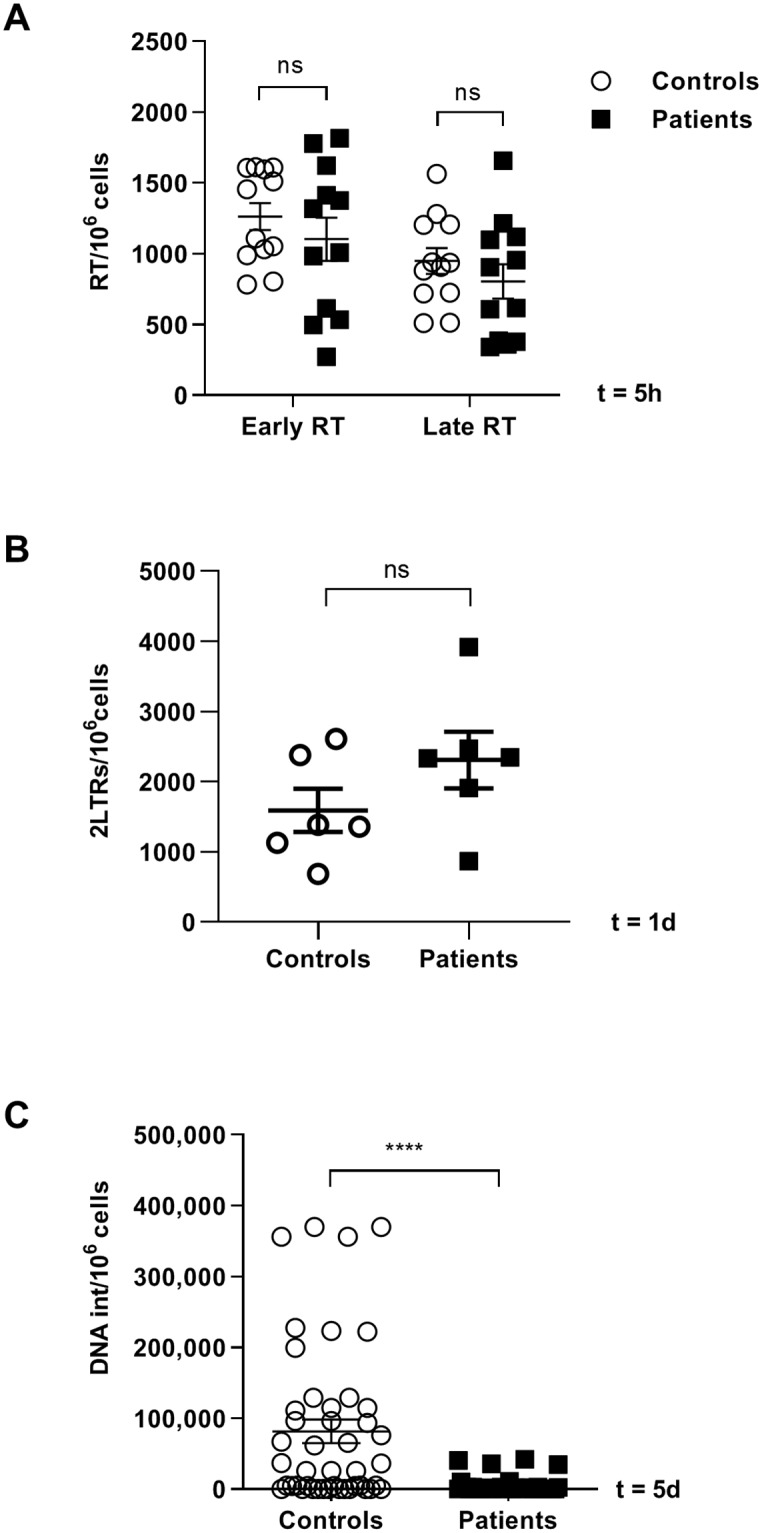
Analysis of different steps of HIV-1 cell cycle in PBMCs from LGMD1F patients and controls. Activated PBMCs of controls and LGMD1F patients were infected with NL4.3-Renilla. **(A)** Analysis by qPCR of early and late reverse transcripts of NL4.3-Renilla in PBMCs infected for 5 hours. **(B)** Analysis by digital PCR of episomal 2-LTR circles 24 hours post-infection. **(C)** Analysis by qPCR of HIV-1 integration after 5 days. All data are represented in a bar graph ± the standard error of the mean (SEM). ns for non-significant. **** p < 0.0001.

PBMCs isolated from twenty patients and twenty-six healthy controls were activated with antiCD3/CD28 and IL-2 for 48 hours and then infected with NL4.3-Renilla by spinoculation. At 5 hours post-infection, DNA was extracted and synthesis of HIV-1 strong stop (R/U5) and full-length (R/ATG-gag) reverse transcriptase products, which represent early and late reverse transcriptase transcripts, respectively, were quantified by qPCR (Fig 4A). No significant difference in the efficiency of reverse transcription in PBMCs from LGMD1F patients and healthy controls was detected.

In order to monitor the nuclear import of viral DNA, the accumulation of circular DNA intermediates was determined by measuring episomal 2-LTRs by ultrasensitive digital PCR 24 hours after infection. No significant difference was detected in the number of episomal 2-LTR circles between PBMCs from LGMD1F patients and healthy controls (Fig 4B).

To assess viral integration, infected cells were incubated for 5 days, DNA was extracted and proviral copies were quantified by qPCR. Proviral integration was on average 16-fold lower in PBMCs from LGMD1F patients than in controls (**** *p*<0.0001) (Fig 4C). These data indicate an integration defect in cells carrying TNPO3 mutation.

### PBMCs from LGMD1F patients showed reduced stability of cytoplasmic HIV-1 PIC after infection

**Fig 5:**
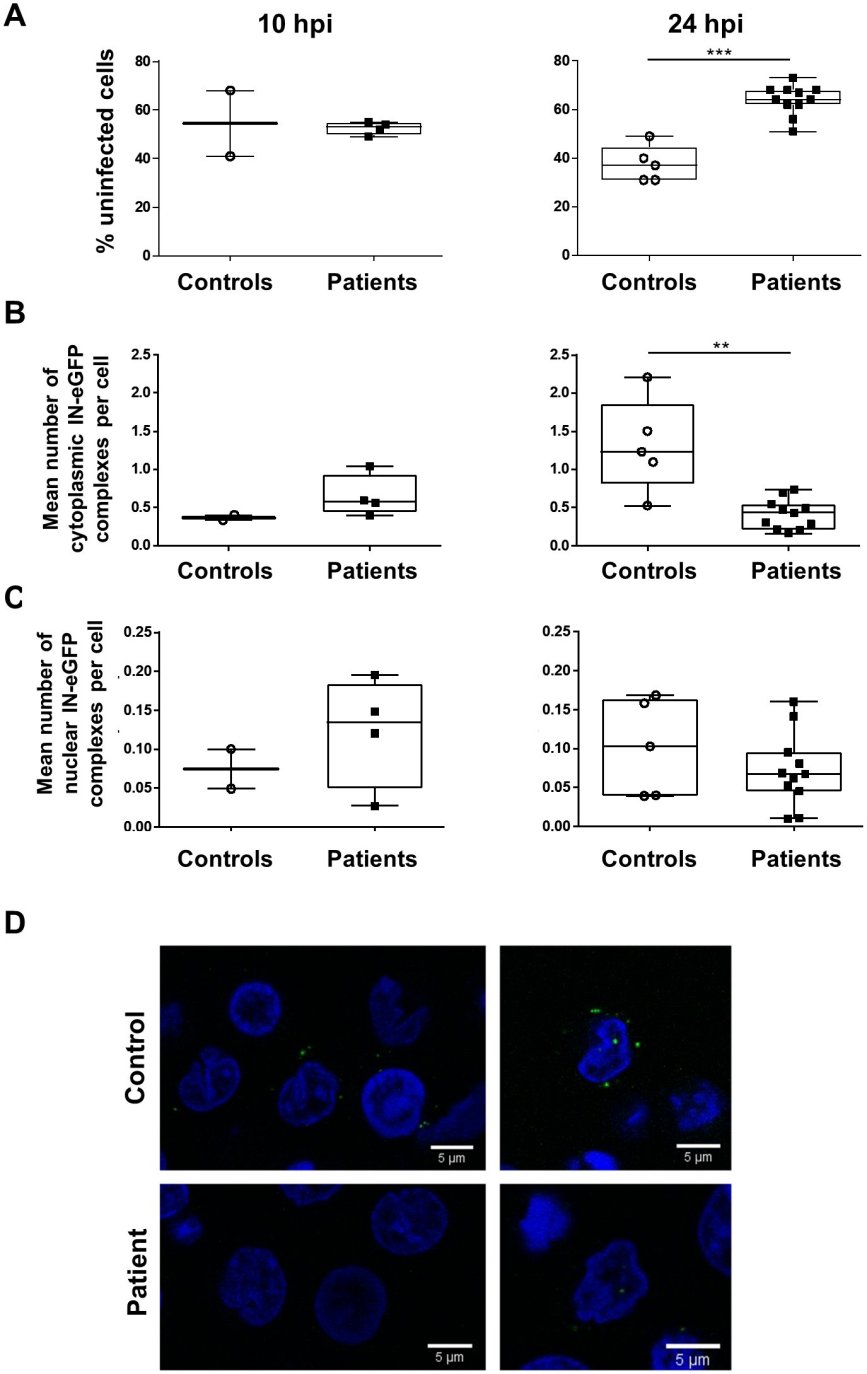
The LGMD1F mutation reduces stability of cytoplasmatic HIV-1 complexes. CD4^+^ T-cells of healthy controls (open circles) and patients with LGMD1F (filled black squares) were infected with HIV_IN-eGFP_. 10 h and 24 h after infection, cells were fixed and the percentage of uninfected cells (**A**), the number of cytoplasmic **(B)** and nuclear IN-eGFP complexes **(C)** were calculated with an in-house MatLab routine. The data of at least two independent experiments were plotted in a scatter plot and a Mann-Whitney test was used to determine statistical significance: ** p < 0.01 and *** p < 0.001. Representative confocal images are shown of lamin (blue) stained CD4+ T cells, from healthy controls (TNPO3_wt) and patients with LGMD1F (TNPO3_mut), infected with HIV^Env^_IN-eGFP_ (green) for 24h. Scale bar represents 5 μm **(D)**.

CD4+ T cells isolated from patients with LGMD1F and healthy controls were infected with fluorescently labeled particles (HIV-IN-eGFP) [7,36]. The presence of IN-eGFP in PICs allows quantitative analysis of the number of PICs and their intracellular location by confocal microscopy. When HIV-1 infected CD4+ T cells were fixed 10 hours post-infection, no significant differences were detected between both groups, neither in the number of uninfected cells (Fig 5A), nor in the number of cytoplasmic and nuclear PICs per cell (Fig 5B and 5C, respectively). However, after 24 hours of infection, the number of cells without PICs was 1.7-fold higher in CD4+ T cells from patients with LGMD1F than in healthy controls (*p*<0.001) (Fig 5A). The number of cytosolic PICs was 3.1-fold reduced in CD4+ T cells from patients with LGMD1F compared to the healthy controls (Fig 5B) (*p*<0.01). This was not due to higher PIC translocation to the nucleus, as there was no significant difference in the number of nuclear PICs (Fig 5C and 5D).

### Validation of TNPO3_mut as host factor refractory to HIV replication

**Fig 6:**
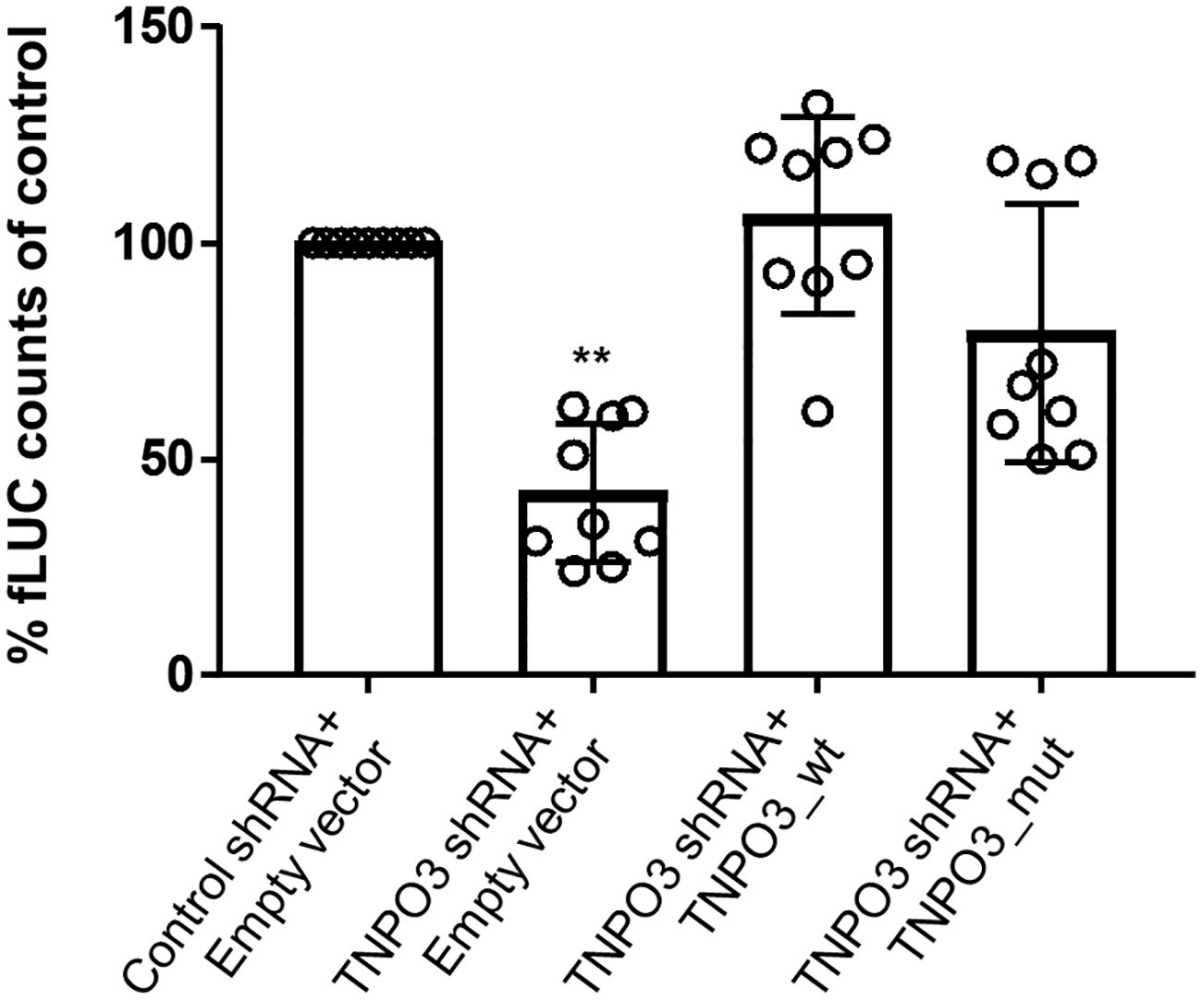
The LGMD1F mutation reduces HIV-1 infectivity. The various transduced HeLaP4 cell lines were infected with HIV-fLuc^VSV-G^. Luciferase activity was measured 72 h post-infection. Data is represented as relative infectivity compared to a cell line expressing endogenous TNPO3 (control shRNA+ empty vector). The data are means of at least two independent experiments and the error bars represent the standard deviation. A Kruskal-Wallis test was used to test for statistical significance: ** p < 0.01.

HIV-1 infectivity was examined in HeLaP4 cell lines stably expressing TNPO3_wt or TNPO3_mut form. These cell lines were validated by western blot, immunocytochemistry and RT-PCR (S4 Fig). Next, we compared the luciferase signal of HIV-fLuc^VSV-G^ in a cell line containing TNPO3_wt which was transduced with an empty vector (control shRNA + empty vector). As described before, depletion of TNPO3 (TNPO3 shRNA + empty vector), resulted in a three-fold drop in luciferase activity, reflecting a decreased viral infectivity (Fig 6) (p<0.01) [7,25]. Back-complementation with TNPO3_wt in stable knock-down cells (TNPO3shRNA + TNPO3_wt) restored the luciferase activity level to that of cell lines containing endogenous TNPO3. Back-complementation with the mutant form of the protein (TNPO3 shRNA+TNPO3_mut) resulted in lower recovery of HIV-1 infection. These experiments supported that TNPO3_mut was not able to rescue HIV-1 replication in these cell lines thus validating its role as a defective host factor impairing HIV infection.

## Discussion

HIV-1 infection remains incurable despite efficient antiretroviral treatments that tackle HIV-1 enzymes and proteins. One potential strategy to develop new therapeutic targets can be based on the study of the interaction between viral proteins and their cellular cofactors. In this regard, TNPO3 and other importins have been previously described as essential cellular proteins for HIV-1 infection [23–25,37]. However the exact mechanism of action of TNPO3 still remains a matter of controversy [38]. Some studies suggest that TNPO3 participates in the nuclear import of PICs [9,25,39,40] whereas other authors propose that TNPO3 promotes HIV-1 infection though the interaction with HIV-1 CA [41–43] or indirectly through the interaction of CPSF6 with HIV-1 CA [12]. Besides an indirect role for TNPO3 in viral integration through its interaction with CA and/or the IN and their respective cellular partners such as CPSF6 or LEDGF/p75 [38] has been proposed. These cellular host factors may affect the nuclear landscape of HIV-1 infection [44] by targeting the viral genome to silent or actively transcribed chromatin [45] opening a new perspective in the mechanisms of HIV-1 latency and reactivation.

Of note, nuclear transport has not been widely studied as a potential target to HIV-1 infection and only recently new hits impeding TNPO3-IN interactions blocking HIV-1 nuclear transport have been described [8]. Besides this role in HIV-1 infection, TNPO3 is also linked to a rare muscular dystrophy termed LGMD1F. The genetic cause for LGMD1F was found to be an adenosine deletion in the stop codon of *TNPO3* gene, which leads to the addition of 15 amino acids to the C-terminal end [30,31]. The effect of this elongation for the functionality of TNPO3 protein is still unknown but previous data indicate that TNPO3_mut shows a perinuclear distribution whereas TNPO3_wt predominantly localizes inside the nucleus. This suggests that TNPO3 mutation could affect the subcellular localization of the protein [31]. Because the cargo-binding domain of TNPO3 resides in the C-terminal end, the additional 15 amino acids may also alter its cargo binding properties and subsequently influence alternative splicing [30,31]. Apart from these preliminary results, the mechanism by which TNPO3 mutation affects its function and causes LGMD1F remains undetermined. Interestingly, other rare muscular diseases known as laminopathies are related to changes in the nuclear lamin architecture, a fibrous structure located below the nuclear membrane which functions include among others the regulation of the nuclear transport [46,47]. Besides, other muscle diseases like myotonic dystrophies are due to mutations in splicing factors. For these reasons the term “splicepathies” has been proposed to design these genetic diseases [48,49]. In summary, the biomedical interest of TNPO3 is first, as an essential cellular protein in the HIV-1 cycle and second through a specific mutation, as the genetic cause of the ultra-rare disease LGMD1F. In this context, we propose that the understanding of HIV-1 biology in lymphocytes from LGMD1F patients represents a tool to get a better insight into the mechanisms of nuclear transport and to understand the pathogenic mechanisms leading to muscle disease such as muscular dystrophies that share common physiopathology pathways.

Our results demonstrate that resistance to HIV-1 infection observed in CD4 lymphocytes from patients with LGMD1F is directly related with the mutation in *TNPO3* gene because the infectivity was not affected when we infected with an HIV-1 clone carrying a CA-mutation (N74D), in which the nuclear transport is independent of TNPO3. As previously described [17], the efficacy of infection is lower with N74D clone as compared to a wt virus, which explains lower luciferase levels. Interestingly, infection with N74D virus was not equal in the PBMCs of all LGMD1F patients as in some of them the infection with the mutant virus was higher than in controls, suggesting that PBMCs from some patients with LGMD1F can develop compensatory mechanism to overcome the deficiency in TNPO3-mediated transport. By unknown reasons, age of onset and severity of clinical symptoms are highly variable in LGMD1F patients, despite all of them carry the same mutation, pointing to the presence of alternative mechanisms that compensate the deficit in TNPO3 function.

Due to the central role of TNPO3 in HIV-1 nuclear import, we hypothesized that HIV-1 infection might be impaired in PBMCs from LGMD1F patients. LGMD1F is an autosomal recessive disease and accordingly, both *TNPO3*_wt and *TNPO3*_mut form are co-expressed in similar quantity. The *TNPO3*_mut allele was expressed and translated as a 15 amino acids longer protein than TNPO3_wt [31]. However despite the presence of 50% of the normal protein, high resistance to HIV-1 infection ex vivo was observed in these cells, with a reduction of more than 18-fold on average in the production of viral proteins. These results suggest that in patients with LGMD1F TNPO3_mut was interfering with the normal function of TNPO3_wt. It has been proposed that TNPO3 multimerizes in order to carry out its nuclear import functions [29,40]. If TNPO3_mut cannot multimerize or interferes with regular multimerization of TNPO3_wt, this could explain the severe restriction of viral infection observed in LGMD1F cells despite the co-dominant expression of both TNPO3_wt and TNPO3_mut.

The cytosolic location of CPSF6 in lymphocytes from patients with LGMD1F confirms the functional impact of the mutated protein “in vivo” for the first time and correlates with previous work showing that TNPO3 knockdown causes CPSF6 to accumulate in the cytoplasm [12]. Relocalization of CPSF6 to the cytosol could decrease its availability to bind to HIV-1 CA and contribute to the increased resistance to infection observed in LGMD1F patients.

To confirm these data we generated HeLaP4 cell lines with stable knock-down of endogenous TNPO3 that were trans-complemented with either TNPO3_wt or TNPO3_mut form associated to LGMD1F. As described before, depletion of endogenous TNPO3 resulted in decreased viral infectivity that was recovered with the back-complementation of TNPO3_wt. However, expression of the TNPO3_mut form of the protein resulted in lower recovery of HIV-1 infection, which supports that the TNPO3_mut form found in LGMD1F patients restricts HIV-1 infection.

In order to define the step at which TNPO3_mut impaired HIV-1 replication, the viral cell cycle was analyzed in PBMCs from LGMD1F patients in comparison with healthy controls. There were no significant differences between the cells of patients and controls in the formation of early and late reverse transcripts 5 hours after the infection. It has been previously described that TNPO3 depletion leads to a reduction in nuclear 2-LTR circles during HIV-1 infection [7,25,50], although other studies did not corroborate these results [10,11,51]. This discrepancy has been attributed to the different primer sets used to detect the intranuclear viral DNA products [12]. In the present study we could not find differences between controls and LGMD1F patients at the level of the formation of episomal 2-LTR circles 24 hours post-infection (Fig 4B). Accordingly, a direct role for TNPO3_mut on the entry of viral DNA into the nucleus cannot be concluded. However, integration was decreased more than 16-fold in PBMCs from LGMD1F patients in comparison to controls 5 days after infection suggesting an impairment of HIV-1 integration due to the TNPO3 defect leading to deep impact on viral replication.

When using fluorescently labeled viruses (HIV-IN-eGFP), no differences were detected in the cellular distribution at 10 hours after infection. However at 24 hours post-infection the number of infected cells, as defined by IN-eGFP detection, was significantly decreased in patients with LGMD1F. Unexpectedly, only cytoplasmic complexes were reduced, suggesting a decreased stability of the complexes that was not a consequence of higher translocation to the nucleus, as there was no increase in the number of nuclear PICs. The change in subcellular distribution of CPSF6 could not explain this observation since an increase in CA stability due to cytosolic CPSF6 location after silencing TNPO3 has been proposed [12]. One potential hypothesis explaining this sharp decrease in cytosolic PIC numbers could be related to the induction of innate immune responses leading to CA degradation. It has been described that in macrophages HIV-1 evades innate immune recognition in macrophages through specific recruitment of cellular factors to the CA, including TNPO3 and CPSF6 to the CA [16]. A deficient binding of TNPO3 and CPSF6 could increase CA sensing and induction of IFN-mediated mechanisms in lymphocytes of LGMD1F patients.

Finally, because integration targeting of the HIV-1 genome to silent or actively transcribed chromatin is linked to nuclear import mechanisms and cellular factors as LEGDF/p75 and CPSF6 we cannot rule out that entry through TNPO3-independent mechanisms in patients with LGMD1F leads to different selection of chromosomal integration sites [52].

Our data confirm for the first time “in vitro” that adequate TNPO3 function is essential for HIV-1 replication infection, but they also provide insights into the role of TNPO3 in LGMD1F. It should be noted that although LGMD1F patients show progressive muscle weakness as the disease advances, they do not show any type of immunodeficiency or higher susceptibility to infectious diseases. Therefore, the presence of TNPO3_mut is not affecting all types of cells in a similar way, being the muscle cells clearly more affected. Moreover, PBMCs from all patients showed similar resistance to HIV-1 infection, while there is a wide variability in the muscular clinical symptoms that affects these patients, although all of them share the same TNPO3 mutation. One possibility that merits further investigation is that TNPO3_mut could be affecting the nuclear import of essential factors for alternative splicing such as SC35 and CPSF6 [15,16]. It has been described that skeletal muscle is the tissue with the highest number of differentially expressed alternative exons [53,54], including isoforms of myogenic transcription factors, metabolic enzymes and myofibrillar proteins [55]. LGMD covers a group of genetically determined disorders that varies depending on factors such as age of onset, rate of disease progression, distribution of muscle weakness and genetic causes. Such array of factors implies different steps of muscle development and neural and hormonal influences that can affect differently the highly complex pattern of muscle-specific transcripts processed by alternative splicing [56]. Therefore, the analysis of muscle cells from LGMD1F patients could determine whether this disease might be caused by defects in TNPO3-mediated import of splicing factors involved in the alternative splicing of essential proteins for muscular contraction.

In conclusion, TNPO3_mut protein expressed in LGMD1F cells is the second genetic defect leading to strong HIV-1 restriction in humans. Importantly, the first genetic defect shown to produce HIV-1 restriction, the CCR5 delta32 deletion, blocks entry of R5-tropic but not X4- tropic strains. *TNPO3* mutation described here acts at a post-entry step in the virus life cycle, and may therefore be independent of viral tropism. These findings increase our understanding of the role of TNPO3 in HIV-1 infection, and support further characterization of LGMD1F as a splicing disease.

## Materials and methods

### Patients and controls

Twenty three patients with diagnosed LGMD1F and twenty-seven healthy donors were recruited for this study. Muscle strength was graded using the Modified Medical Research Council (MMRC) scale. The upper and lower extremity functions were assessed using Brooke and Vignos scores, respectively [34,35]. LGMD1F patients were recruited at the Hospital de La Fe (Valencia, Spain) and healthy donors were recruited at the Centro Regional de Transfusión from the Complejo Hospitalario de Toledo (Toledo, Spain).

### Ethics statement

All individuals gave informed written consent and this study was approved by the Institutional Ethical Committee Board of Hospital de La Fe (2016/0388) and Instituto de Salud Carlos III (Madrid, Spain; CEI PI 22_2017-v3).

### Quantitative PCR for allelic discrimination

Total genomic DNA and RNA were isolated from PBMCs using DNA/RNA Mini Kit (Qiagen). SNP genotyping assay was designed for detecting simultaneously *TNPO3*_wt and *TNPO3*_mut forms using the following primers and probes: forward primer (5´-GCGAGACTTCACCAGGTTGTT-3´); reverse primer (5´-CTGGGTGACAGGCACAGT-3´); TNPO3_wt probe: (TNPO3 deletion-V, VIC, 5´-CAGGAGTGTGAGCTATCGA-3´); and TNPO3_mut probe (TNPO3 deletion-M, FAM, 5´-AGGAGTGTGAGCATCGA-3´). cDNA was synthesized from 200 ng of RNA by using GoScript Reverse Transcription System (Promega), following manufacturers’ instructions. RT cycling conditions were as follows: 5 min at 25°C; 1h at 45°C; and 15 min at 70°C. SNP genotyping was also performed using 50 ng of genomic DNA and TaqMan Universal Mix (Applied Biosystems). Analyses were performed in triplicate per sample using StepOne Real-Time PCR system (Applied Byosistems) with standard cycling conditions. Results for the allelic discrimination were represented as ΔRn, being Rn the ratio between the fluorescent emission intensity of the reporter dye and the passive dye.

### Immunoblotting assays

Whole protein extracts were obtained as described previously [57] and protein concentration was determined by Bradford method [58]. Ten micrograms of protein extracts were fractionated by SDS-PAGE and transferred onto Hybond-ECL nitrocellulose paper (GE Healthcare). Subsequently, the membranes were blocked and incubated with an anti-TNPO3 antibody (Abcam). Analysis was performed by chemiluminescence using a BioRad Geldoc 2000 (Bio-Rad Laboratories, Madrid, Spain).

### HIV-1 infection

Infectious supernatants were obtained from calcium phosphate transfection of HEK293T cells (provided by the existing collection of the Instituto de Salud Carlos III, Madrid, Spain) with plasmid pNL4.3-Renilla, which contains the HIV-1 proviral clone pNL4.3 with the *nef* gene replaced by *renilla luciferase* gene. The pNL4.3_N74D-Renilla clone was generated introducing the N74D mutation in the nucleotide position 1405 of the previously described plasmid pNL4.3-Renilla [59]. PBMCs were isolated from blood samples by centrifugation using a Ficoll-Hypaque gradient (GE Healthcare) and then activated for 3 days with purified anti-human CD3 (clone OKT3), CD28 (clone CD28.2) (Biosciences, San Diego, CA) and 300 U/ml interleukin-2 (IL-2) (Chiron, Emeryville, CA). Activated cells were infected with 1 ng p24 of NL4.3-Renilla or NL4.3_N74D-Renilla per million of cells by spinoculation. Briefly, after 30 minutes of gently rotation at room temperature, cells were centrifuged at 600xg for 30min at 25°C and extensively washed with PBS1X. Infected cells were cultured for 5 days with IL-2. Renilla activity (RLU) was quantified at different time points in the cell lysates with Renilla Luciferase Assay System (Promega) and measured with a Sirius luminometer (Berthold Detection Systems, Oak Ridge, TN). Data were normalized for protein concentration measured with the Bradford method [58] and cell viability was measured with the CellTiter-Glo Luminescent Cell Viability assay (Promega).

### Quantification of early and late reverse transcription

At 5 hours after infection, DNA was extracted using QIAamp DNA Blood Mini Kit (Qiagen) and quantified with Nanodrop 2000C (Thermo Scientific). Strong stop DNA was quantified using primer pairs specific for R and U5 regions of the HIV-1 long terminal repeat (LTR), as described [60]. Serial dilutions of genomic DNA from 8E5 cell line, which contains a single integrated copy of HIV-1 [61], were used as a standard curve. The *ccr5* gene was used as endogenous control. qPCR was performed in triplicate in StepOne Real-Time PCR system (Applied Biosystems) using standard cycling conditions.

### Quantification of episomal 2-LTR circles by digital PCR

Genomic DNA was extracted from PBMCs 24 hours after infection with NL4.3-Renilla, using DNeasy Blood and Tissue kit (Qiagen), and quantified using the Nanodrop-1000 spectrophotometer (Nanodrop). The samples were measured by the QuantStudio 3D Digital PCR System (Life Technologies). The reaction mixture for Digital PCR (dPCR) is as follows: cDNA, QuantStudio 3D Digital PCR Master Mix v2, 300nM of C1_2LTR primer, 300nM C4R_71 primer, 250nM 2nr4nr_FAM probe, 0,5x CCR5_VIC probe and H_2_O for a final volume of 14.5 μl. The digital PCR reaction mix was loaded onto the QuantStudio 3D digital PCR chips, according to the manufacturer instructions. The thermal cycling and amplifications were performed in a ProFlex 2xFlat PCR system, according to the manufacturer protocol: 96°C for 10 min, followed 39 cycles of 55 °C for 2 min and 98°C for 30 sec, 55 °C for 2 min and a stabilization phase at 22°C. The chips were transferred to a QuantStudio 3D digital PCR instrument for imaging and the final analysis of the files generated was carried out using the cloud-based.

### Quantification of proviral integration by TaqMan qPCR

Total DNA from PBMCs infected for 5 days with NL4.3-Renilla was extracted using DNeasy Blood and Tissue kit (Qiagen) as described above and the integrated HIV-DNA was measured by nested Alu–HIV-LTR PCR [62–64] with modifications [65]. Briefly, 50 ng DNA were used for the first conventional PCR (C1000 Thermal Cycler, Bio-Rad) with 10x TaqMan Buffer, dNTPs, Alu-1 primer, Alu-2 primer, L-M667 primer and Platinum Taq DNA polymerase (Roche); 2x TaqMan Universal PCR Master (Promega), AA55M primer, Lambda T primer and MH603 probe for the second quantitative PCR (StepOne, Applied Biosystems). A standard curve of integrated HIV-DNA from 8E5 cell line using serial dilutions was prepared as reference and CCR5 was used as housekeeping gene.

### Analysis of HIV-1 PIC

HIV-1 particles containing fluorescently labeled IN (HIV-IN-eGFP) were generated by Vpr-mediated trans-incorporation as described before [7,36,66]. For infection with HIV-IN-eGFP, PBMCs were previously incubated with CD3:CD28 bispecific monoclonal antibody (NIH AIDS Reagent Program, Division of AIDS, NIH from Drs. Johnson Wong and Galit Alter) during 5 days for CD4+ T cell enrichment. CD4+ T cells were infected with HIV-IN-eGFP by spinoculation. At 2 hours post-infection cells were washed and further incubated for 5 or 19 hours. Next, the cells were plated in poly-D-lysine chambers and allowed to adhere, to reach a total infection time of 10 h and 24 h, respectively. Cells were fixed for 15 min with 4% (v/v) paraformaldehyde and permeabilized during 5 min with 0.1% (v/v) Triton-X100. Nuclei were immunostained with lamin A/C antibody (Santa Cruz Biotechnology) and secondary anti-Mouse IgG (H+L) Alexa Fluor 555 conjugate (ThermoFisher Scientific) diluted in blocking buffer (1% (w/v) BSA and 0.1% (v/v) Tween-20 in PBS). Imaging of the cells was performed using a laser scanning microscope (Fluoview FV1000, Olympus, Tokyo, Japan). An in-house MatLab routine (MatWorks) was used to determine the localization and number of IN-eGFP complexes [7]. In short, IN-eGFP complexes and the nuclear lamin were determined automatically using an intensity threshold based on the triangle algorithm. Based on the nuclear lamin staining, IN-eGFP complexes were divided into cytoplasmic or nuclear compartments and the percentage of nuclear IN-eGFP complexes was calculated. Typically, data were collected from 90 cells.

### Infection of stably transduced HeLaP4 cell lines with HIV-fLuc^VSV-G^

Vesicular stomatitis virus glycoprotein (VSV-G) pseudotyped lentiviral vectors for the stable expression of TNPO3 were produced as described before [7,25]. In short, 6.5x10^6^ HEK-293T cells were transfected with 10 μg of the packaging construct pCMVδR8.91 [67], 20 μg transfer construct (pCHMWS-3xFLAG-TNPO3-IRES-hygro with TNPO3_wt or TNPO3_mut) and 5 μg pVSV-G using branched polyethylenimine (bPEI, 10 μM, Sigma-Aldrich). Supernatant was collected 48 h and 72 h post-transfection, filtered through a 0.45 μm pore-size filter, and concentrated by ultrafiltration (Amicon Ultra-15 Centrifugal Filter Unit, 50 kDa, Merck).

HeLaP4 cells, a kind gift from Dr. P. Charneau (Institut Pasteur, France), and HeLaP4 cells stably depleted of TNPO3 [7,25] were back-complemented with TNPO3_wt or TNPO3_mut through stable transduction with lentiviral vectors. Briefly, 3x10^4^ HeLaP4 cells were plated in a 96-well plate the day before transduction. The next day, cells were transduced with a dilution series of vector containing cassettes coding for TNPO3_wt or TNPO3_mut. As a control, cells were transduced with an empty cassette vector (empty vector). Transduced cells were selected with 160 μg/ml hygromycin.

To determine the effect of the TNPO3_mut on the viral infectivity in cell lines, 1.5x10^4^ HeLaP4 cells were seeded per well in a 96-well plate. The next day, the cells were infected with a three-fold dilution of a single-round HIV-fLuc^VSV-G^ [68,69]. At 72 h post-infection, cells were lysed in buffer (50 mM Tris, 200 mM NaCl, 0.2% (v/v) NP40 and 5% (v/v) glycerol) and analysed for firefly luciferase activity (ONE-Glo Promega GMBH, Mannheim, Germany). Chemiluminescence was measured with a Glomax luminometer (Promega). Readouts were normalised for protein content as determined by a BCA protein assay. Data are represented as relative infectivity compared to a cell line expressing endogenous TNPO3_wt (control shRNA) and are means of at least two independent experiments. A Kruskal-Wallis test was used to evaluate statistical significance. Error bars represent the standard deviation.

### Statistical analysis

Statistical analysis was performed using GraphPad Prism 5.0 Software (GraphPad). Comparisons between LGMD1F patients and healthy individuals were made with one-way analysis of variance (ANOVA) using Tukey´s Multiple Comparison Test to describe statistical differences among groups. The number of nuclear and cytoplasmic PIC was plotted in a scatter plot and a Mann-Whitney test was used to determine statistical significance. Differences were considered statistically significant when ** p < 0.01, *** p < 0.001 and **** p < 0.0001.

## Supporting information

S1 Fig Expression of surface and activation markers. Analysis by flow cytometry of the expression of surface markers CD4, CXCR4 and CCR5 in resting PBMCs from seven LGMD1F patients and four healthy controls (**A**) and the expression of activation markers CD25 and HLA-DR in PBMCs from four LGMD1F patients and two healthy controls (**B**). Cells were stained with monoclonal antibodies conjugated with fluorochromes and then analyzed in FACS Calibur cytometer (Becton Dickinson Biosciences) using CellQuest software. Data are represented using Graphpad Prism 7 software.
(TIF)

S2 Fig Representative confocal microscopy images of intracellular expression of CPSF6. PBMCs of LGMD1F patients and controls were activated for 3 days with purified anti-CD3, anti-CD28 and IL-2. Intracellular expression was confirmed by immunofluorescence using a monoclonal antibody against CPSF6 and a secondary antibody conjugated to Alexa 488 (green). DAPI was used for nuclear staining (blue). The results are representative of those observed in four independent patients and four controls. Bars indicate 5 μm.
(TIF)

S3 Fig Survival of infection in vitro. Activated PBMCs of controls and LGMD1F patients were infected with NL4.3-Renilla (**A**) and NL4.3_N74D-Renilla (**B**) to follow the kinetics of viral infection. Viability in infected cells were measured at 1,2,3,4 and 7 days with the CellTiter-Glo Luminescent Cell Viability assay (Promega).
(TIF)

S4 Fig Validation of TNPO3 expressing cell lines. HeLaP4 cell lines expressing endogenous TNPO3 (control shRNA and control shRNA + empty vector) or depleted of TNPO3 (TNPO3 shRNA and TNPO3 shRNA + empty vector) were back-complemented with lentiviral vectors encoding either FLAG-TNPO3_wt (TNPO3 shRNA + TNPO3_wt) or FLAG-TNPO3_mut (TNPO3 shRNA + TNPO3_mut and control shRNA + TNPO3_mut). (**A**) Expression levels were determined by western blot analysis with anti-TNPO3 antibody. β-tubulin was included as a loading control. (**B**) Fluorescence microscopy images of cells stained with anti-FLAG antibody (red). Scale bar: 10 μm. (**C**) The mRNA levels were determined b RT-qPCR. Error bars represent the standard deviation.
(TIF)

S1 File Materials and methods. (DOCX)

## References

[1] Coiras M, López-Huertas MR, Del Cojo MS, Mateos E, Alcamí J. Dual Role of Host Cell Factors in HIV-1 Replication: Restriction and Enhancement of the Viral Cycle. AIDS Reviews. 2010 pp. 103–112. 20571604

[2] Loetscher P, Moser B, Baggiolini M. Chemokines and Their Receptors in Lymphocyte Traffic and HIV Infection. 1999 pp. 127–180. 10.1016/s0065-2776(08)60910-4 10605606

[3] Piller S, Caly L, Jans D. Nuclear Import of the Pre-Integration Complex (PIC): The Achilles Heel of HIV? Curr Drug Targets. 2005;4: 409–429. 10.2174/1389450033490984 12816349

[4] Farnet CM, Bushman FD. HIV-1 cDNA integration: Requirement of HMG I(Y) protein for function of preintegration complexes in vitro. Cell. 1997;88: 483–492. 10.1016/s0092-8674(00)81888-7 9038339

[5] Shun MC, Raghavendra NK, Vandegraaff N, Daigle JE, Hughes S, Kellam P, et al LEDGF/p75 functions downstream from preintegration complex formation to effect gene-specific HIV-1 integration. Genes Dev. 2007;21: 1767–1778. 10.1101/gad.1565107 17639082 PMC1920171

[6] De Rijck J, Vandekerckhove L, Christ F, Debyser Z. Lentiviral nuclear import: A complex interplay between virus and host. BioEssays. 2007 pp. 441–451. 10.1002/bies.20561 17450594

[7] Borrenberghs D, Dirix L, De Wit F, Rocha S, Blokken J, De Houwer S, et al Dynamic Oligomerization of Integrase Orchestrates HIV Nuclear Entry. Sci Rep. 2016;6 10.1038/srep36485 27830755 PMC5103197

[8] Demeulemeester J, Blokken J, Houwer S, Dirix L, Klaassen H, Marchand A, et al Inhibitors of the integrase-transportin-SR2 interaction block HIV nuclear import. Retrovirology. 2018;15 10.1186/s12977-018-0389-2 29329553 PMC5767004

[9] Tsirkone VG, Blokken J, De Wit F, Breemans J, De Houwer S, Debyser Z, et al N-terminal half of transportin SR2 interacts with HIV integrase. J Biol Chem. 2017; 10.1074/jbc.M117.777029 28356354 PMC5465493

[10] Zhou L, Sokolskaja E, Jolly C, James W, Cowley SA, Fassati A. Transportin 3 promotes a nuclear maturation step required for efficient HIV-1 integration. PLoS Pathog. 2011;7 10.1371/journal.ppat.1002194 21901095 PMC3161976

[11] Valle-Casuso JC, Di Nunzio F, Yang Y, Reszka N, Lienlaf M, Arhel N, et al TNPO3 Is Required for HIV-1 Replication after Nuclear Import but prior to Integration and Binds the HIV-1 Core. J Virol. 2012;86: 5931–5936. 10.1128/JVI.00451-12 22398280 PMC3347269

[12] De Iaco A, Santoni F, Vannier A, Guipponi M, Antonarakis S, Luban J. TNPO3 protects HIV-1 replication from CPSF6-mediated capsid stabilization in the host cell cytoplasm. Retrovirology. 2013;10 10.1186/1742-4690-10-20 23414560 PMC3599327

[13] Lai MC, Lin RI, Huang SY, Tsai CW, Tarn WY. A human importin-β family protein, transportin-SR2, interacts with the phosphorylated RS domain of SR proteins. J Biol Chem. 2000;275: 7950–7957. 10.1074/jbc.275.11.7950 10713112

[14] Lai MC, Kuo HW, Chang WC, Tarn WY. A novel splicing regulator shares a nuclear import pathway with SR proteins. EMBO J. 2003;22: 1359–1369. 10.1093/emboj/cdg126 12628928 PMC151058

[15] Lai M-C, Lin R-I, Tarn W-Y. Transportin-SR2 mediates nuclear import of phosphorylated SR proteins. Proc Natl Acad Sci. 2001;98: 10154–10159. 10.1073/pnas.181354098 11517331 PMC56931

[16] Kataoka N, Bachorik JL, Dreyfuss G. Transportin-SR, a nuclear import receptor for SR proteins. J Cell Biol. 1999;145: 1145–1152. 10.1083/jcb.145.6.1145 10366588 PMC2133142

[17] Rasaiyaah J, Tan CP, Fletcher AJ, Price AJ, Blondeau C, Hilditch L, et al HIV-1 evades innate immune recognition through specific cofactor recruitment. Nature. 2013;503: 402–405. 10.1038/nature12769 24196705 PMC3928559

[18] Ocwieja KE, Sherrill-Mix S, Mukherjee R, Custers-Allen R, David P, Brown M, et al Dynamic regulation of HIV-1 mRNA populations analyzed by single-molecule enrichment and long-read sequencing. Nucleic Acids Res. 2012;40: 10345–10355. 10.1093/nar/gks753 22923523 PMC3488221

[19] Dowling D, Nasr-Esfahani S, Tan CH, O’Brien K, Howard JL, Jans DA, et al HIV-1 infection induces changes in expression of cellular splicing factors that regulate alternative viral splicing and virus production in macrophages. Retrovirology. 2008;5: 18 10.1186/1742-4690-5-18 18241354 PMC2267807

[20] Hallay H, Locker N, Ayadi L, Ropers D, Guittet E, Branlant C. Biochemical and NMR study on the competition between proteins SC35, SRp40, and heterogeneous nuclear ribonucleoprotein A1 at the HIV-1 Tat exon 2 splicing site. J Biol Chem. 2006;281: 37159–37174. 10.1074/jbc.M603864200 16990281

[21] Jacquenet S, Decimo D, Muriaux D, Darlix JL. Dual effect of the SR proteins ASF/SF2, SC35 and 9G8 on HIV-1 RNA splicing and virion production. Retrovirology. 2005;2 10.1186/1742-4690-2-33 15907217 PMC1180853

[22] Ropers D, Ayadi L, Gattoni R, Jacquenet S, Damier L, Branlant C, et al Differential effects of the SR proteins 9G8, SC35, ASF/SF2, and SRp40 on the utilization of the A1 to A5 splicing sites of HIV-1 RNA. J Biol Chem. 2004;279: 29963–29973. 10.1074/jbc.M404452200 15123677

[23] Brass AL, Dykxhoorn DM, Benita Y, Yan N, Engelman A, Xavier RJ, et al Identification of host proteins required for HIV infection through a functional genomic screen. Science (80). 2008;319: 921–926. 10.1126/science.1152725 18187620

[24] König R, Zhou Y, Elleder D, Diamond TL, Bonamy GMC, Irelan JT, et al Global analysis of host-pathogen interactions that regulate early-stage HIV-1 replication. Cell. 2008;135: 49–60. 10.1016/j.cell.2008.07.032 18854154 PMC2628946

[25] Christ F, Thys W, De Rijck J, Gijsbers R, Albanese A, Arosio D, et al Transportin-SR2 Imports HIV into the Nucleus. Curr Biol. 2008;18: 1192–1202. 10.1016/j.cub.2008.07.079 18722123

[26] Diaz-Griffero F. The Role of TNPO3 in HIV-1 Replication. Mol Biol Int. 2012;2012: 1–6. 10.1155/2012/868597 22888429 PMC3409535

[27] Bushby K. Diagnosis and management of the limb girdle muscular dystrophies. Practical Neurology. 2009 pp. 314–323. 10.1136/jnnp.2009.193938 19923111

[28] Gamez J, Navarro C, Andreu AL, Fernandez JM, Palenzuela L, Tejeira S, et al Autosomal dominant limb-girdle muscular dystrophy: A large kindred with evidence for anticipation. Neurology. 2001;56: 450–454. 10.1212/wnl.56.4.450 11222786

[29] Maertens GN, Cook NJ, Wang W, Hare S, Gupta SS, Oztop I, et al Structural basis for nuclear import of splicing factors by human Transportin 3. Proc Natl Acad Sci. 2014;111: 2728–2733. 10.1073/pnas.1320755111 24449914 PMC3932936

[30] Melià MJ, Kubota A, Ortolano S, Vílchez JJ, Gámez J, Tanji K, et al Limb-girdle muscular dystrophy 1F is caused by a microdeletion in the transportin 3 gene. Brain. 2013;136: 1508–1517. 10.1093/brain/awt074 23543484 PMC3634201

[31] Torella A, Fanin M, Mutarelli M, Peterle E, Del Vecchio Blanco F, Rispoli R, et al Next-Generation Sequencing Identifies Transportin 3 as the Causative Gene for LGMD1F. PLoS One. 2013;8 10.1371/journal.pone.0063536 23667635 PMC3646821

[32] Samson M, Libert F, Doranz BJ, Rucker J, Liesnard C, Farber M, et al Resistance to HIV-1 infection in caucasian individuals bearing mutant alleles of the CCR-5 chemokine receptor gene. Nature. 1996;382: 722–726. 10.1038/382722a0 8751444

[33] Liu R, Paxton WA, Choe S, Ceradini D, Martin SR, Horuk R, et al Homozygous defect in HIV-1 coreceptor accounts for resistance of some multiply-exposed individuals to HIV-1 infection. Cell. 1996;86: 367–377. 10.1016/s0092-8674(00)80110-5 8756719

[34] Brooke JD, Hoare J, Rosenrot P, Triggs R. Computerized system for measurement of force exerted within each pedal revolution during cycling. Physiol Behav. 1981; 10.1016/0031-9384(81)90090-1 7232509

[35] VIGNOS PJ, WARNER JL. Glycogen, Creatine, and High Energy Phosphate in Human Muscle Disease. J Lab Clin Med. 1963;62: 579–590. Available: http://www.ncbi.nlm.nih.gov/pubmed/14080857 14080857

[36] Albanese A, Arosio D, Terreni M, Cereseto A. HIV-1 pre-integration complexes selectively target decondensed chromatin in the nuclear periphery. PLoS One. 2008;3 10.1371/journal.pone.0002413 18545681 PMC2398779

[37] Bushman FD, Malani N, Fernandes J, D’Orso I, Cagney G, Diamond TL, et al Host cell factors in HIV replication: Meta-analysis of genome-wide studies. PLoS Pathogens. 2009 10.1371/journal.ppat.1000437 19478882 PMC2682202

[38] Hilditch L, Towers GJ. A model for cofactor use during HIV-1 reverse transcription and nuclear entry. Current Opinion in Virology. 2014 10.1016/j.coviro.2013.11.003 24525292 PMC3969716

[39] De Houwer S, Demeulemeester J, Thys W, Rocha S, Dirix L, Gijsbers R, et al The HIV-1 integrase mutant R263A/K264A is 2-fold defective for TRN-SR2 binding and viral nuclear import. J Biol Chem. 2014; 10.1074/jbc.M113.533281 25063804 PMC4155696

[40] Larue R, Gupta K, Wuensch C, Shkriabai N, Kessl JJ, Danhart E, et al Interaction of the HIV-1 intasome with transportin 3 protein (TNPO3 or TRN-SR2). J Biol Chem. 2012;287: 34044–34058. 10.1074/jbc.M112.384669 22872640 PMC3464514

[41] Krishnan L, Matreyek KA, Oztop I, Lee K, Tipper CH, Li X, et al The Requirement for Cellular Transportin 3 (TNPO3 or TRN-SR2) during Infection Maps to Human Immunodeficiency Virus Type 1 Capsid and Not Integrase. J Virol. 2010; 10.1128/jvi.01899-09 19846519 PMC2798409

[42] Yamashita M, Emerman M. Capsid Is a Dominant Determinant of Retrovirus Infectivity in Nondividing Cells. J Virol. 2004; 10.1128/jvi.78.11.5670-5678.2004 PMC415837 15140964

[43] Yamashita M, Emerman M. The cell cycle independence of HIV infections is not determined by known karyophilic viral elements. PLoS Pathog. 2005; 10.1371/journal.ppat.0010018 16292356 PMC1283251

[44] Lusic M, Siliciano RF. Nuclear landscape of HIV-1 infection and integration. Nature Reviews Microbiology. 2017 10.1038/nrmicro.2016.162 27941817

[45] Achuthan V, Perreira JM, Sowd GA, Puray-Chavez M, McDougall WM, Paulucci-Holthauzen A, et al Capsid-CPSF6 Interaction Licenses Nuclear HIV-1 Trafficking to Sites of Viral DNA Integration. Cell Host Microbe. 2018; 10.1016/j.chom.2018.08.002 30173955 PMC6368089

[46] Broers JL V., Ramaekers FCS, Bonne G, Ben Yaou R, Hutchison CJ. Nuclear Lamins: Laminopathies and Their Role in Premature Ageing. Physiol Rev. 2006;86: 967–1008. 10.1152/physrev.00047.2005 16816143

[47] Brull A, Rodriguez BM, Bonne G, Muchir A, Bertrand AT. The pathogenesis and therapies of striated muscle laminopathies. Frontiers in Physiology. 2018 10.3389/fphys.2018.01533 30425656 PMC6218675

[48] Caillet-Boudin M-L, Fernandez-Gomez F-J, Tran H, Dhaenens C-M, Buee L, Sergeant N. Brain pathology in myotonic dystrophy: when tauopathy meets spliceopathy and RNAopathy. Front Mol Neurosci. 2014;6 10.3389/fnmol.2013.00057 24409116 PMC3885824

[49] Udd B, Krahe R. The myotonic dystrophies: Molecular, clinical, and therapeutic challenges. The Lancet Neurology. 2012 pp. 891–905. 10.1016/S1474-4422(12)70204-1 22995693

[50] Thys W, De Houwer S, Demeulemeester J, Taltynov O, Vancraenenbroeck R, Gérard M, et al Interplay between HIV Entry and Transportin-SR2 Dependency. Retrovirology. 2011;8 10.1186/1742-4690-8-7 21276267 PMC3041740

[51] De Iaco A, Luban J. Inhibition of HIV-1 infection by TNPO3 depletion is determined by capsid and detectable after viral cDNA enters the nucleus. Retrovirology. 2011;8: 98 10.1186/1742-4690-8-98 22145813 PMC3267670

[52] Ocwieja KE, Brady TL, Ronen K, Huegel A, Roth SL, Schaller T, et al HIV integration targeting: A pathway involving transportin-3 and the nuclear pore protein RanBP2. PLoS Pathog. 2011;7 10.1371/journal.ppat.1001313 21423673 PMC3053352

[53] Pan Q, Shai O, Lee LJ, Frey BJ, Blencowe BJ. Deep surveying of alternative splicing complexity in the human transcriptome by high-throughput sequencing. Nat Genet. 2008;40: 1413–1415. 10.1038/ng.259 18978789

[54] Wang ET, Sandberg R, Luo S, Khrebtukova I, Zhang L, Mayr C, et al Alternative isoform regulation in human tissue transcriptomes. Nature. 2008;456: 470–6. 10.1038/nature07509 18978772 PMC2593745

[55] Ladd AN, Charlet-B N, Cooper TA. The CELF Family of RNA Binding Proteins Is Implicated in Cell-Specific and Developmentally Regulated Alternative Splicing. Mol Cell Biol. 2002;21: 1285–1296. 10.1128/mcb.21.4.1285-1296.2001 PMC99581 11158314

[56] Pistoni M, Ghigna C, Gabellini D. Alternative splicing and muscular dystrophy. RNA Biology. 2010 pp. 441–452. 10.4161/rna.7.4.12258 20603608 PMC3568746

[57] Laín de Lera T, Folgueira L, Martín AG, Dargemont C, Pedraza M-A, Bermejo M, et al Expression of IκBα in the nucleus of human peripheral blood T lymphocytes. Oncogene. 1999;18: 1581–1588. 10.1038/sj.onc.1202455 10102628

[58] Bradford MM. A rapid and sensitive method for the quantitation of microgram quantities of protein utilizing the principle of protein-dye binding. Anal Biochem. 1976;72: 248–254. 10.1006/abio.1976.9999 942051

[59] Garcia-Perez J, Sanchez-Palomino S, Perez-Olmeda M, Fernandez B, Alcami J. A new strategy based on recombinant viruses as a tool for assessing drug susceptibility of human immunodeficiency virus type 1. J Med Virol. 2007;79: 127–137. 10.1002/jmv.20770 17177310

[60] Mohammadi P, Desfarges S, Bartha I, Joos B, Zangger N, Muñoz M, et al 24 Hours in the Life of HIV-1 in a T Cell Line. PLoS Pathog. 2013;9 10.1371/journal.ppat.1003161 23382686 PMC3561177

[61] Folks TM, Powell D, Lightfoote M, Koenig S, Fauci AS, Benn S, et al Biological and biochemical characterization of a cloned Leu-3- cell surviving infection with the acquired immune deficiency syndrome retrovirus. J Exp Med. 1986;164: 280–90. Available: http://www.ncbi.nlm.nih.gov/pubmed/3014036%0Ahttp://www.pubmedcentral.nih.gov/articlerender.fcgi?artid=PMC2188205 3014036 10.1084/jem.164.1.280 PMC2188205

[62] Butler SL, Hansen MST, Bushman FD. A quantitative assay for HIV DNA integration in vivo. Nat Med. 2001;7: 631–634. 10.1038/87979 11329067

[63] Brussel A, Sonigo P. Analysis of Early Human Immunodeficiency Virus Type 1 DNA Synthesis by Use of a New Sensitive Assay for Quantifying Integrated Provirus. J Virol. 2003;77: 10119–10124. 10.1128/JVI.77.18.10119-10124.2003 12941923 PMC224570

[64] Dismuke DJ, Aiken C. Evidence for a Functional Link between Uncoating of the Human Immunodeficiency Virus Type 1 Core and Nuclear Import of the Viral Preintegration Complex. J Virol. 2006;80: 3712–3720. 10.1128/JVI.80.8.3712-3720.2006 16571788 PMC1440469

[65] Bermejo M, López-Huertas MR, Hedgpeth J, Mateos E, Rodríguez-Mora S, Maleno MJ, et al Analysis of protein kinase C theta inhibitors for the control of HIV-1 replication in human CD4+ T cells reveals an effect on retrotranscription in addition to viral transcription. Biochem Pharmacol. 2015;94: 241–256. 10.1016/j.bcp.2015.02.009 25732195

[66] Borrenberghs D, Thys W, Rocha S, Demeulemeester J, Weydert C, Dedecker P, et al HIV virions as nanoscopic test tubes for probing oligomerization of the integrase enzyme. ACS Nano. 2014;8: 3531–3545. 10.1021/nn406615v 24654558 PMC4004294

[67] Zufferey R, Nagy D, Mandel RJ, Naldini L, Trono D. Multiply attenuated lentiviral vector achieves efficient gene delivery in vivo. Nat Biotechnol. 1997;15: 871–875. 10.1038/nbt0997-871 9306402

[68] Connor RI, Chen BK, Choe S, Landau NR. Vpr is required for efficient replication of HIV-1.pdf. Virology. 1995 pp. 935–44. 7531918 10.1006/viro.1995.1016

[69] Jowett JB, Planelles V, Poon B, Shah NP, Chen ML, Chen IS. The human immunodeficiency virus type 1 vpr gene arrests infected T cells in the G2 + M phase of the cell cycle. J Virol. 1995;69: 6304–13. 7666531 10.1128/jvi.69.10.6304-6313.1995 PMC189529

