# Supplementary figures and images for: The mutation of Transportin 3 gene that causes limb girdle muscular dystrophy 1F induces protection against HIV-1 infection
Source: PLoS Pathog. 2019 Aug 29;15(8):e1007958. doi: 10.1371/journal.ppat.1007958 (PMC6715175; doi:10.1371/journal.ppat.1007958)

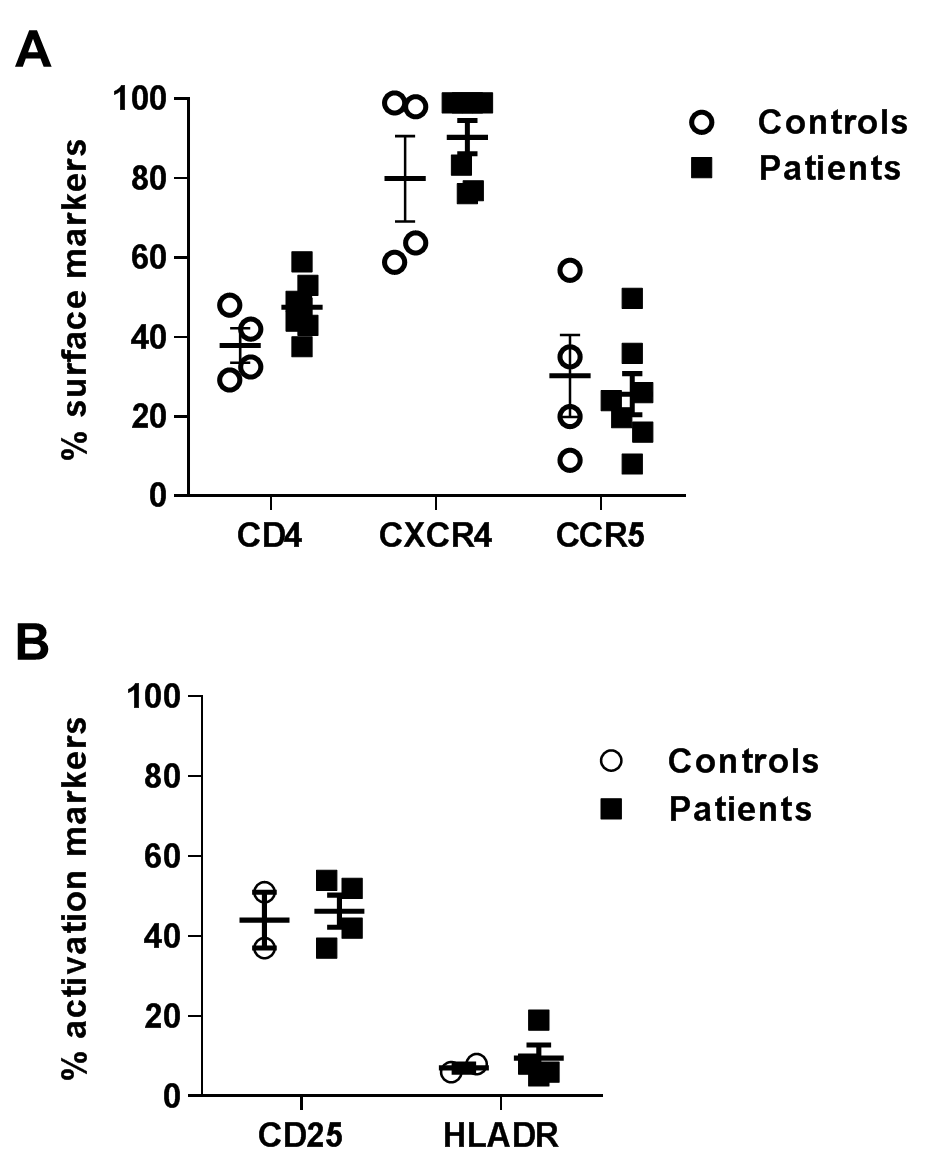

Supplement: S1 Fig — Analysis by flow cytometry of the expression of surface markers CD4, CXCR4 and CCR5 in resting PBMCs from seven LGMD1F patients and four healthy controls (A) and the expression of activation markers CD25 and HLA-DR in PBMCs from four LGMD1F patients and two healthy controls (B). Cells were stained with monoclonal antibodies conjugated with fluorochromes and then analyzed in FACS Calibur cytometer (Becton Dickinson Biosciences) using CellQuest software. Data are represented using Graphpad Prism 7 software. (TIF) [file ppat.1007958.s001.tif]

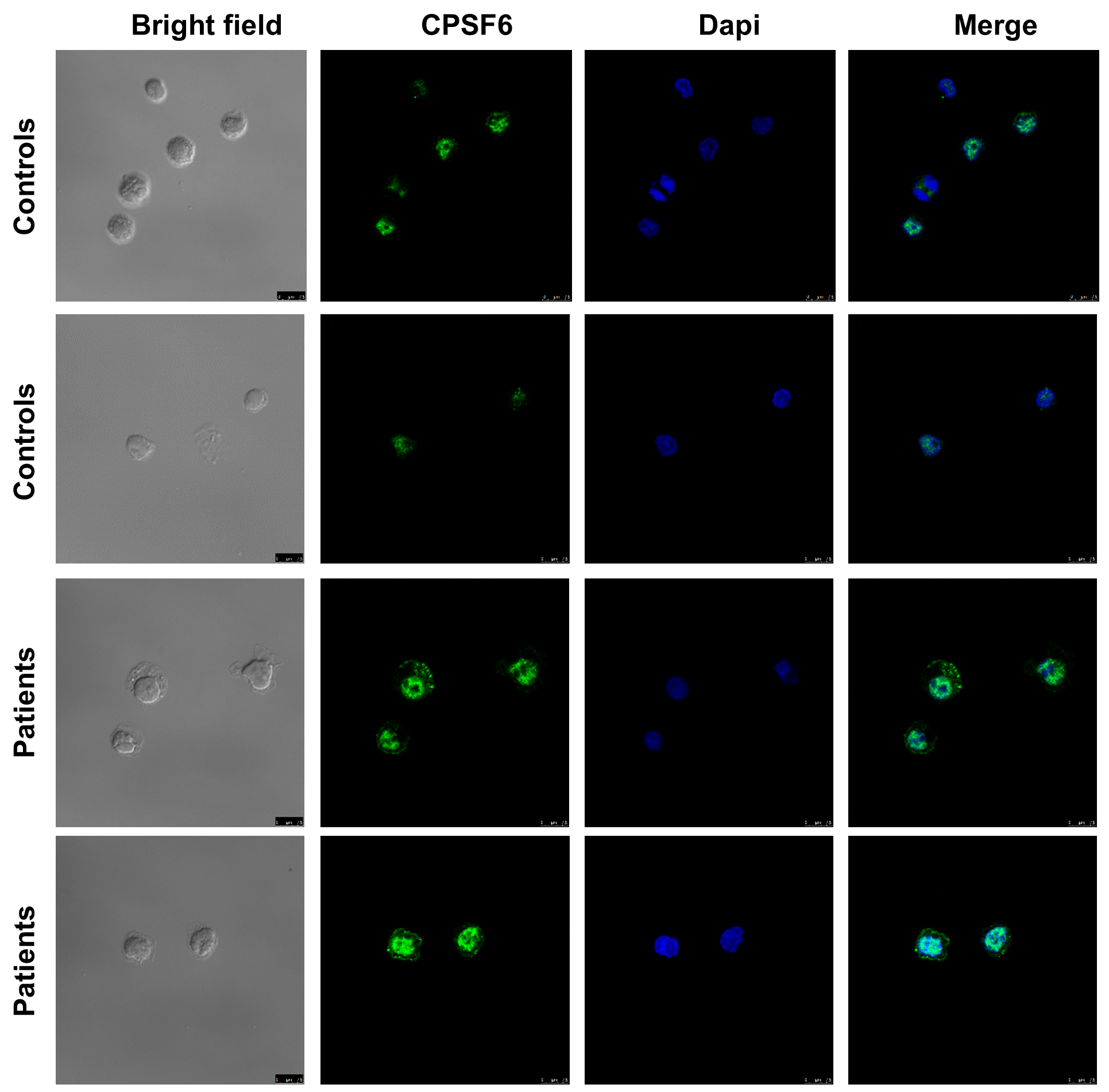

Supplement: S2 Fig — PBMCs of LGMD1F patients and controls were activated for 3 days with purified anti-CD3, anti-CD28 and IL-2. Intracellular expression was confirmed by immunofluorescence using a monoclonal antibody against CPSF6 and a secondary antibody conjugated to Alexa 488 (green). DAPI was used for nuclear staining (blue). The results are representative of those observed in four independent patients and four controls. Bars indicate 5 μm. (TIF) [file ppat.1007958.s002.tif]

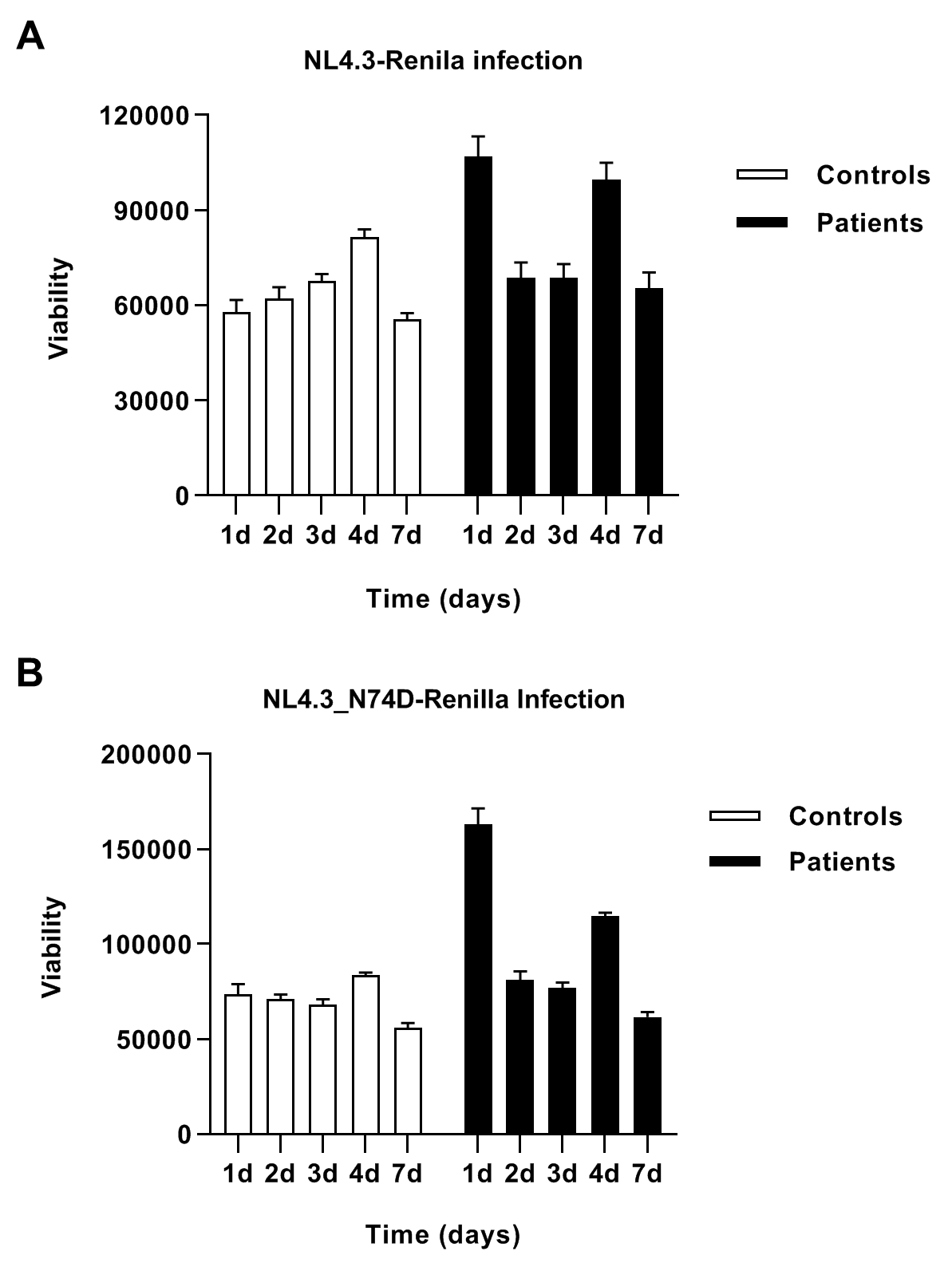

Supplement: S3 Fig — Activated PBMCs of controls and LGMD1F patients were infected with NL4.3-Renilla (A) and NL4.3_N74D-Renilla (B) to follow the kinetics of viral infection. Viability in infected cells were measured at 1,2,3,4 and 7 days with the CellTiter-Glo Luminescent Cell Viability assay (Promega). (TIF) [file ppat.1007958.s003.tif]

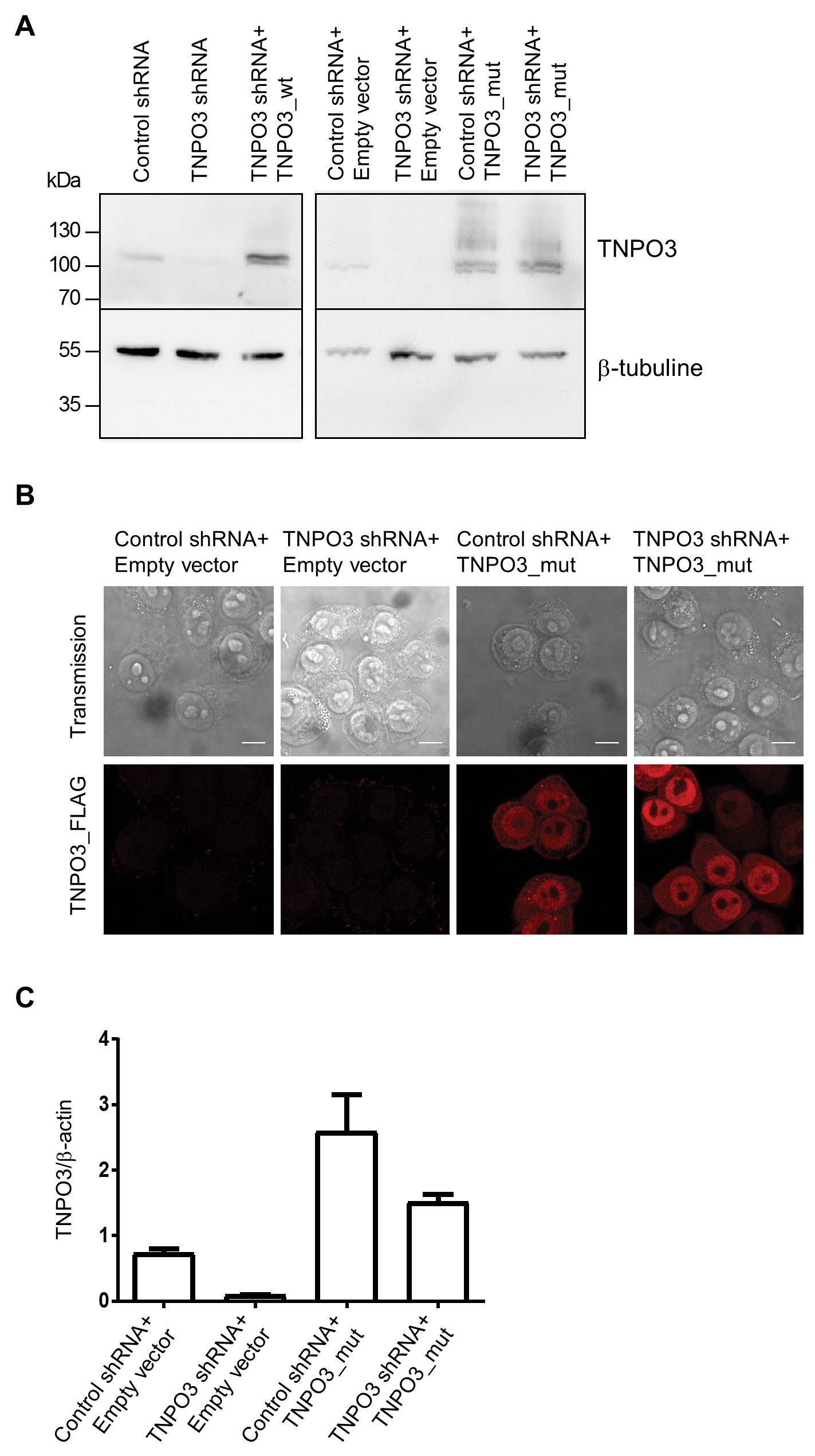

Supplement: S4 Fig — HeLaP4 cell lines expressing endogenous TNPO3 (control shRNA and control shRNA + empty vector) or depleted of TNPO3 (TNPO3 shRNA and TNPO3 shRNA + empty vector) were back-complemented with lentiviral vectors encoding either FLAG-TNPO3_wt (TNPO3 shRNA + TNPO3_wt) or FLAG-TNPO3_mut (TNPO3 shRNA + TNPO3_mut and control shRNA + TNPO3_mut). (A) Expression levels were determined by western blot analysis with anti-TNPO3 antibody. β-tubulin was included as a loading control. (B) Fluorescence microscopy images of cells stained with anti-FLAG antibody (red). Scale bar: 10 μm. (C) The mRNA levels were determined b RT-qPCR. Error bars represent the standard deviation. (TIF) [file ppat.1007958.s004.tif]
