# Supplementary material for: The mutation of Transportin 3 gene that causes limb girdle muscular dystrophy 1F induces protection against HIV-1 infection
Source: PLoS Pathog. 2019 Aug 29;15(8):e1007958. doi: 10.1371/journal.ppat.1007958 (PMC6715175; doi:10.1371/journal.ppat.1007958)
Supplement: S1 File — (DOCX) [file ppat.1007958.s005.docx]

**S1 Materials and Methods**

**Flow cytometry analysis.** 2x10^5^ PBMCs from controls and LGMD1F patients were washed with 1xPBS and stained with fluorescent labeled monoclonal antibodies against surface markers conjugated (CD4-PerCP, CXCR4-PE and CCR5-FITC, BD Biosciences) or activation markers (CD25-PE and HLADR-FITC, BD Biosciences). After 15 min incubation, the cells were washed twice with PBS1X and then fixed with 1% paraformaldehyde in 1xPBS for 1 h. Data acquisition was performed in a FACScalibur Flow Cytometer. Data analysis was done using CellQuest software.

**Immunofluorescence Assays.** PBMCs of two LGMD1F patients and two controls activated for three 3 days with purified anti-CD3, anti-CD28 and IL-2 were immobilized on PolyPrep slides (Sigma-Aldrich) for 15 min and then fixed with 2% paraformaldehyde (PFA) in 1× PBS for 10 min at room temperature. After washing twice with 1xPBS, cells were permeabilized with 0.1% Triton X-100/PBS. The samples were washed with 1xPBS–2% BSA–0.05% saponine buffer and incubated with the primary antibody anti-CPSF6 (Santa Cruz Biotechlogy). After subsequent washes with 1xPBS–2% BSA–0.05% saponine buffer, the samples were incubated with secondary antibody conjugated to Alexa 488 (Thermo Fisher Scientific) and 4′,6-diamidino-2-phenylindole (Dapi, Sigma) was used for nuclear staining. Images were obtained with Leica TCS-SP confocal microscope (Leica Microsystems, Wetzlar, Germany).

**Cell Viability.** Cell viability was determined using the CellTiter-Glo Luminescent Cell Viability assay (Promega) following the manufacturer's instructions. Briefly, 1x10^5^ cells were harvested by centrifugation, washed twice with 1xPBS, and resuspended in lysis buffer. After incubation for 10 min at room temperature to stabilize luminescent signal, cell lysates were deposited in an opaque-walled multiwell plate and analyzed in an Orion Microplate Luminometer with Simplicity software (Berthold Detection Systems, Oak Ridge, TN).

**Immunoblot assays.** Protein concentrations of 1% (w/v) SDS whole cell lysates were determined using a bicinchoninic acid (BCA) protein assay (BCA Protein Assay Kit, Thermo Scientific). For Western blotting, 30 μg of the cell lysate was directly loaded onto a 12.5% (w/v) sodium-dodecyl sulphate polyacrylamide gel electrophoresis (SDS-PAGE) gel and electroblotted onto polyvinylidene difluoride membranes (PVDF, BioRad). TNPO3 detection was done using antibodies against TNPO3 (mouse monoclonal, 1/100 dilution, ab54353, Abcam). Equal loading of cell lysates was confirmed with β-tubulin antibody (mouse monoclonal, 1/1000 dilution, T4026, Sigma). Blots were subsequently stained with a horseradish peroxidase-conjugated secondary antibody (Dako) and detected by chemiluminescence (ECL+, BioRad) with a Las-3000 Mini (FujiFilm).

**RT-qPCR for quantification mRNA levels in HeLaP4 cells.** To determine the TNPO3 mRNA levels of TNPO3-depleted and TNPO3-back-complemented cells, RNA was extracted from 2x10^6^ HeLaP4 cells using the Aurum total RNA Mini Kit (BioRad) and 25 ng was reverse transcribed into cDNA using the High Capacity cDNA Reverse Transcription Kit (Applied Biosystems). TNPO3 or β-actin mRNA was amplified using the iQ Supermix (BioRad) and in-house designed primers and probes. Each reaction contained 12.5 µl iQ Supermix, 5 µl cDNA, 100 nM or 200 nM forward and primer, 100 nM or 200 nM probe and water in a total volume of 25 µl for the detection of β-actin or TNPO3 mRNA, respectively. Each sample was run in triplicate for 3 minutes at 95°C followed by 50 cycles of 10 seconds at 95°C and 30 seconds at 55°C using the iQ5 Multicolor RT PCR Detection system and iQ5 Optical System software (BioRad). The TNPO3 mRNA levels were normalised for β-actin content.

**Immunocytochemistry for TNPO3.** To determine the TNPO3 localization in the transduced cells, 3x10^4^ HeLaP4 cells were seeded per well in poly-D-lysine (Sigma-Aldrich) coated 8-well chambered coverglasses (Nunc Lab-Tek Chambered Coverglasses, ThermoFisher Scientific). After 6 h, cells were fixed with 4% (v/v) paraformaldehyde for 15 min, permeabilised with 0.1% (v/v) Triton for 5 min, and blocked with blocking buffer (phosphate buffered saline (PBS), 0.1% (v/v) Tween (Applichem) and 1% (w/v) BSA) for 1 h. Afterwards, the cells were immunostained with anti-FLAG primary antibody (mouse monoclonal, 1/400 dilution, F3165, Sigma) and secondary anti-Mouse IgG (H+L) Alexa Fluor 633 conjugate (goat polyclonal, 1/1000 dilution, ThermoFisher Scientific) diluted in blocking buffer. Imaging of the cells was performed using a laser scanning microscope.
